# Fidaxomicin Reduces Collagen Expression in Intestinal Fibroblasts Via Platelet-Derived Growth Factor Receptor Beta and Glycogen Synthase Kinase-3 Beta Inhibition

**DOI:** 10.1053/j.gastro.2025.04.028

**Published:** 2025-05-19

**Authors:** Sophie Irwin, Mieke van Daelen, Isabella Almaraz, Rebecca Park, Becca Nelson, Swapna Mahurkar-Joshi, Florian Rieder, David Q. Shih, Wendy Ho, Berkeley Limketkai, Hon Wai Koon

**Affiliations:** 1 Vatche and Tamar Manoukian Division of Digestive Diseases, David Geffen School of Medicine at the University of California Los Angeles, Los Angeles, California;; 2 Goodman-Luskin Microbiome Center, David Geffen School of Medicine at UCLA, Los Angeles, California;; 3 F. Widjaja Foundation, Inflammatory Bowel & Immunobiology Research Institute, Cedars-Sinai Medical Center, Los Angeles, California;; 4 Department of Gastroenterology, Hepatology, and Nutrition, Digestive Diseases Institute, Department of Inflammation and Immunity, Lerner Research Institute Program for Global Translational Inflammatory Bowel Diseases, Cleveland Clinic Foundation, Cleveland, Ohio

**Keywords:** Fibrosis, Receptor, High-Throughput Screening, Spatial RNA Sequencing, Protein Array

## Abstract

**BACKGROUND & AIMS:**

About 30%–50% of patients with Crohn’s disease (CD) eventually develop intestinal strictures, with intestinal fibrosis being a major component of them. There is currently no approved medication to treat fibrotic strictures.

**METHODS:**

10X Genomics Visium spatial RNA sequencing and high-throughput screening were used to discover the molecular targets of intestinal fibrosis. Stricturing Crohn’s disease (CDS) patient-derived primary human intestinal fibroblasts (CD-HIFs), stricturing Crohn’s disease patient-derived serum exosomes (CDSE), fresh surgically resected whole-thickness ileal tissues, and mouse models of intestinal fibrosis were used.

**RESULTS:**

Spatial RNA sequencing found overexpression of platelet-derived growth factor receptor beta (PDGFRB) in the fibrotic ileal tissues of CDS patients. PDGFRB small interfering RNA inhibited collagen expression in the CDSE-treated CD-HIFs. High-throughput screening identified PDGFRB inhibitors that suppressed collagen promoter activity in CDSE-treated CD-HIFs. A machine learning algorithm and molecular docking predicted PDGFR as a target for fidaxomicin. Fidaxomicin, a Food and Drug Administration–approved drug for *Clostridioides difficile* infection, inhibited collagen and PDGFRB messenger RNA (mRNA) expression in CDSE-treated CD-HIFs and CDS patient-derived ileal tissues. CDSE-treated CD-HIFs had increased PDGFR*β* and glycogen synthase kinase-3 alpha/beta (GSK3ɑ*/β*) phosphorylation. Fidaxomicin inhibited PDGFR*β* phosphorylation, PDGFRB mRNA expression, and GSK3*β* phosphorylation in CDSE-treated CD-HIFs. The anti-fibrogenic effect of fidaxomicin was attenuated by platelet-derived growth factor-BB (PDGF-BB) and insulin-like growth factor 1, which are a PDGFR*β* ligand and a GSK3ɑ/*β* phosphorylation activator, respectively. In the SAMP1/YitFc mice, oral fidaxomicin treatment inhibited ileal fibrosis and ileal PDGFRB mRNA expression and PDGFR*β* and GSK3*β* phosphorylation, which were abolished by Pdgfrb and Gsk3b overexpression.

**CONCLUSIONS:**

Fidaxomicin inhibits intestinal fibrosis by reducing PDGFRb phosphorylation and expression, GSK3b phosphorylation, and collagen expression in intestinal fibroblasts.

## Graphical Abstract

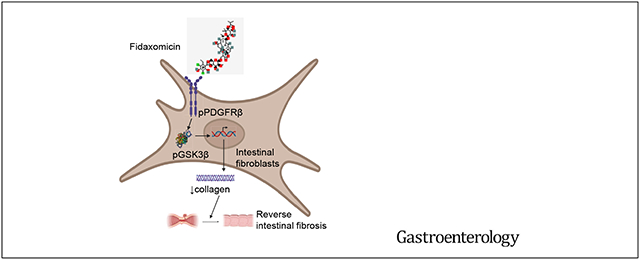

Crohn’s disease (CD) currently affects around 0.3% of Americans.^1^ Approximately 30%–50% of CD patients may eventually develop an intestinal stricture, ie, with fibrosis of the intestine being a major component of it.^2,3^ Intestinal fibrosis is characterized by excessive extracellular matrix (ECM) deposition, especially collagen. The excessive ECM deposition obstructs bowel movement. Intestinal strictures often lead to hospitalization and surgery. Endoscopic balloon dilation and strictureplasty can some-times alleviate mild short-segment bowel narrowing, but surgical resection may be needed to resolve more severe and complex bowel obstructions.^4^ Surgical resection is the last resort because it adversely affects the patient’s quality of life. While some anti-inflammatory drugs, such as anti-tumor necrosis factor agents, may be helpful in reducing symptomatic CD strictures,^5^ there is no pharmacologic agent that can treat purely fibrotic strictures.^6^ For this reason, antifibrogenic therapy is sought to avoid surgery.

Host responses participate in intestinal fibrosis development.^6–11^ Recently, new technologies have been available to find new stricture-related genes with improved precision. Intestinal strictures develop unevenly in the intestines of CD patients.^12^ To implement effective, safe, and specific therapies for treating intestinal fibrosis, it is imperative to identify stricture-related genes within the fibrotic areas of ileal tissues in CDS patients and discover new antifibrogenic drugs.

We hypothesize that our multi-omics target discovery approach, combined with novel drug screening and bioinformatics workflow, will help identify ECM-regulating antifibrogenic agents, specifically targeting stricture-related genes in intestinal fibroblasts. This study aimed to identify a Food and Drug Administration (FDA)–approved drug for modulating an ECM-regulating gene and inhibiting intestinal fibrosis.

## Methods

### Human Blood Samples and Ileal Tissues

Human blood samples were prospectively collected from the University of California Los Angeles (UCLA) from 2012–2023 from remnants of medically indicated blood collections. The UCLA Institutional Review Board (12–001499) approved this study and waived separate informed consent because UCLA Pathology obtained written informed consent from all subjects. Serum samples were pooled from stricturing Crohn’s disease (CDS) patients, and serum exosomes (CDSE) were prepared with a total exosome isolation reagent (4478360, ThermoFisher).^11,13^

To isolate CDS patient-derived peripheral blood mononuclear cells (CDS-PBMCs), ethylenediaminetetra-acetic acid–treated whole blood was incubated with ACK lysing buffer (A1049201, ThermoFisher) in a 1:10 ratio for 5 minutes to remove red blood cells. This was followed by centrifugation at 800*g* for 5 minutes at room temperature. After the removal of cell-free supernatants, the pellets were resuspended in RPMI1640 with 10% exosome-depleted fetal bovine serum (A2720803, ThermoFisher) for experiments.

Baseline characteristics of blood donors are shown in Supplementary Table 1.

Fresh, surgically resected, full-thickness ileal tissues from CDS patients with fibrotic morphology were prospectively collected from UCLA Surgical Pathology during 2021–2023. The UCLA Institutional Review Board (12–001499) approved the study. Fresh human intestinal tissues were cut into 3 × 3 mm and incubated in serum-free RPMI1640 (11875119, ThermoFisher) with 100 mg/mL CDSE to simulate the CD environment and induce fibrogenesis.^13^

Medical notes of ileal tissue donors are shown in Supplementary Table 2.

Inclusion criteria were as follows: CD patients with and without intestinal strictures, as diagnosed by UCLA gastroenterologists, using clinical evaluation, imaging modalities, and/or ileocolonoscopy.

Exclusion criteria were as follows: pregnant women, prisoners, minors younger than age 18 years, and patients with concurrent acute infection (cytomegalovirus infection, *Clostridioides difficile [C difficile]* infection [CDI], and tuberculosis) and malignant conditions.

#### Cell Culture

CDS patient-derived primary human intestinal fibroblasts (CD-HIFs) were cultured in fibroblast media (M2267, Cell Biologics).^11,13^ CDS patient-derived primary human intestinal epithelial cells (HPECs) were cultured in epithelial cell media (H6621, Cell Biologics).^13,14^ The baseline characteristics of the donors are shown in Supplementary Table 3. When the CD-HIFs and HPECs reached confluence, they were then cultured in serum-free Dulbecco’s Modified Eagle Medium (DMEM; 11965092, ThermoFisher). Serum-starved CD-HIFs/HPECs were pretreated with 100 mg/mL CDSE to simulate the CD environment and induce fibrogenesis.^13^

At the end of the experiments, the cells were lysed with radioimmunoprecipitation assay buffer (89900, ThermoFisher) containing protease and phosphatase inhibitors (A32959, ThermoFisher) for enzyme-linked immunosorbent assay (ELISA).

Pro-collagen I alpha 1/ProCOL1A1 (DY6220–05), phosphorylated platelet-derived growth factor receptor beta/PDGFR*β* (DYC1767), and phosphorylated glycogen synthase kinase-3 alpha/beta/GSK3ɑ/*β* (DYC2630) proteins in cell and tissue lysates were measured using ELISA from R&D Systems. Phosphorylated GSK3ɑ (PEL-GSK3ɑ-S21–1) and GSK3*β* (PEL-GSK3*β*-S9–1) proteins in cell and tissue lysates were measured using ELISA from RayBiotech.

For RNA extraction, the cells were lysed with RLT buffer from Qiagen RNeasy kits. Whole transcriptome RNA sequencing and bioinformatic analyses were performed at the UCLA Technology Center for Genomics and Bioinformatics.^15^

### High-Throughput Screening of Human Endogenous Metabolites

CD-HIFs in 96-well plates (165305, Thermo Scientific) were transfected with 5 μg/plate collagen 1A1 (COL1A1) promoter-mCherry construct (HPRM30073-PM02, GeneCopoeia) via 5 *μ*L/well Opti-MEM (31985091, ThermoFisher), 0.3 *μ*L/well Lipofectamine 3000 (L3000008, ThermoFisher), and 100 *μ*L/well serum-free DMEM overnight. The transfected CD-HIFs were subsequently treated with 100 μg/mL CDSE to mimic the CD environment and stimulate fibrogenesis.^13^ Thirty minutes later, 2621 FDA-approved drugs from a drug screening library (HY-L030, MedChemExpress) were added to the plates at a final concentration of 10 *μ*M.^15^ After 24 hours, CD-HIFs were stained with 1 *μ*L/well Hoechst 33342 nuclear stain (R37605, ThermoFisher). An Agilent automated Lion-heart LX imager was used to capture the fluorescence signals from Texas red and 4’,6-diamidino-2-phenylindole blue channels, and images were analyzed using Agilent Gen5+ software. The relative ratio of COL1A1 promoter activity (Texas red channel) over the Hoechst nuclear (4’,6-diamidino-2-phenylindole channel) signal was calculated. Drugs with relative COL1A1 promoter activity inhibition by more than 2 standard deviations (SDs) were identified as hits.

The same COL1A1 promoter mCherry-Hoechst 33342 assays were also used to assess collagen promoter activities in the validation study of fidaxomicin and stricture-related genes in CD-HIFs and HPECs.

### Animal Experiments

UCLA Institutional Animal Research Committee (2007–116) approved the animal studies. All methods were compliant with the ARRIVE (Animal Research: Reporting of In Vivo Experiments) guidelines.^16^ Animal facility staff randomized and blindly assigned mice to cages. The mice were housed under standard environmental conditions in the UCLA animal facility. All interventions were performed during the light cycle. The male-to-female ratio was 1:1.

### Spontaneous CD-Like Ileal Fibrosis in Mice

The SAMP1/YitFc mice (009355, Jackson Laboratories) develop spontaneous CD-like chronic ileitis with pre-existing ileal fibrosis around 40 weeks of age.^17^ Forty-week-old AKR mice (000648, Jackson Laboratories) served as a parental nonfibrotic control strain.^18^ Mice, at 40 weeks of age, were intraperitoneally injected with Pdgfrb-overexpressing (LV7–36306064, abmgood), Gsk3b-overexpressing (LV7–22736064, abmgood), and Gsk3b-small interfering RNA (siRNA) (iLV7–22736094, abmgood) lentiviruses or an anti-PDGFR*β* neutralizing antibody (16-1402-82, Invitrogen). Ileal tissues were collected for analyses at 42 weeks of age.^13^ The experiments were carried out in 4 rounds. Samples of the 8 groups were processed together for assays.

### Power Analysis

Three mice per group were required to achieve statistical power to detect differences in ileal fibrosis scores between 42-week-old SAMP1/YitFc mice with and without fidaxomicin (2.8 vs 0.7) with SD = 0.9, alpha = 05, and power = 0.8. We did not perform power analysis for cell culture experiments but followed the common practice of performing in vitro experiments 3–4 times independently.

### Statistical Analysis

Results were expressed as mean ± SD. Two-group comparisons were performed with unpaired Student *t* tests, and multiple-group comparisons were performed with 1-way analysis of variance (ANOVA) (GraphPad Prism 10). The *P* values of statistical significance are shown in each figure or table.

### SAGER Guidelines

We followed SAGER guidelines. This study covered both genders. Male and female mice were used in a 1:1 ratio. Sex in human cells and tissues was reported in the baseline characteristics tables.

## Results

### Spatial RNA Sequencing Discovered Common Fibrosis-Related Genes in the Fibrotic Regions of the Ileal Tissues of CDS Patients

**Figure 1.**
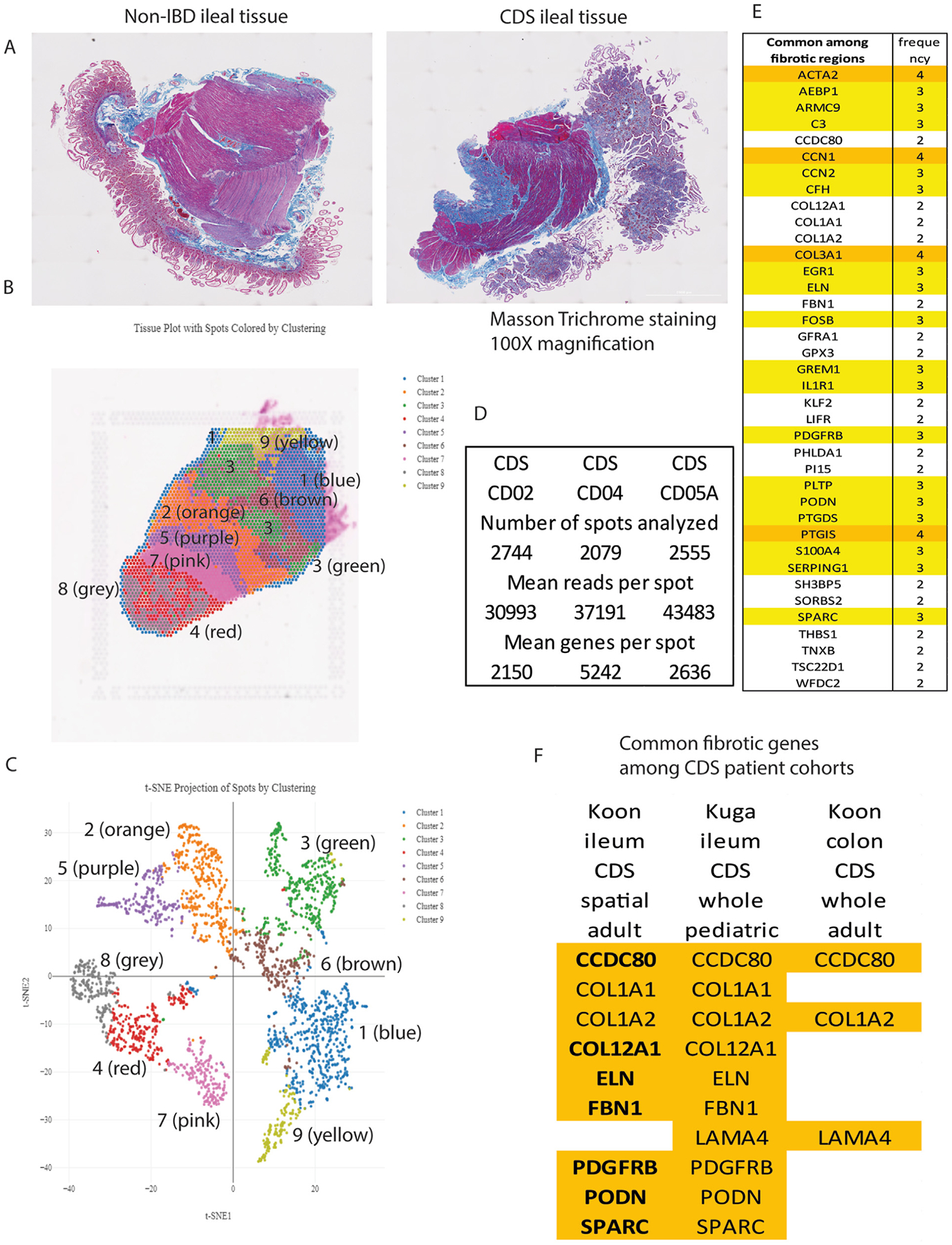
Spatial RNA sequencing revealed commonly overexpressed genes in the fibrotic regions of intestinal strictures in CDS patients. (*A*) Masson’s trichrome staining of fibrotic ileal tissues from a CDS (CD05A) patient and nonfibrotic ileal tissues from a non-IBD (CD06A) patient. The fibrotic tissue has more intense collagen deposition (as indicated by *blue color*) than the nonfibrotic tissue. Scale bars of 2000 *μ*m are in the lower right corner. (*B–E*) 10X Genomics Visium spatial RNA sequencing of the ileal tissues of CDS patients. (*B*) Spatial clustering of a piece of ileal tissue from a CDS patient (CD02). (*C*) t-SNE (t-distributed stochastic neighbor embedding) projection of spots by clustering to show the differentiation of data among various clusters from a piece of ileal tissue from a CDS patient (CD02). (*D*) The performance data of 10X Genomics Visium spatial RNA sequencing of 3 ileal tissues of CDS patients was satisfactory. (*E*) The consistently up-regulated genes in the 5 fibrotic clusters of ileal tissues from these 3 CDS patients. The frequency of overexpressed genes >2 of 5 fibrotic clusters is shown. (*F*) A comparison of up-regulated genes in ileal tissues in CDS patients, including spatial RNA sequencing of ileal tissues from our adult CD patient cohort (comparing fibrotic and nonfibrotic regions in the same CDS patients), a whole-transcriptome RNA sequencing of ileal tissues from a pediatric CD patient cohort (comparing B2 fibrostenosing vs B1 inflammatory phenotype),^7^ and a whole-transcriptome RNA sequencing of colonic tissues (comparing CDS vs CDNS patients) from our previously reported adult CD patient cohort.^13^

This study focuses on fibrotic ileal strictures. The fibrotic ileal tissues from CDS patients have more intense collagen deposition than the nonfibrotic ileal tissues from non-IBD patients (Figure 1A). Fibrosis occurs unevenly in the intestine of CDS patients. Therefore, we performed 10X Genomics Visium spatial RNA sequencing to compare the gene signature in the fibrotic regions vs adjacent less-fibrotic regions. Different gene expression patterns defined multiple clusters in the CDS patient-derived ileal explants (Figure 1B and C). The sample performance metrics met the sequencing requirement of the Visium spatial gene expression (Figure 1D). Five fibrotic clusters from 3 CDS patients were identified based on significantly higher collagen messenger RNA (mRNA) expression than other clusters in the same explant (Supplementary Table 4). By comparison of 5 fibrotic clusters in the ileal tissues, 37 genes were consistently up-regulated in at least 2 fibrotic clusters (Figure 1E).

### Colonic and Ileal Fibrosis Share Some Common Stricture-Related Genes in CDS Patients

Comparing ileal spatial RNA sequencing data with whole-transcriptome RNA sequencing of pediatric CD patients’ ileal tissues and the whole-transcriptome RNA sequencing of adult CD patients’ colonic tissues,^7,11^ ileal and colonic strictures showed different patterns of up-regulated genes (Figure 1F). Collagen 1A2 (COL1A2) is commonly up-regulated in all cohorts. Elastin and fibrillin 1 are common ileal ECMs. Coiled-coil domain-containing 80 (CCDC80) and laminin subunit alpha 4 (LAMA4) are common among pediatric ileal and adult colonic strictures. PDGFRB, podocan (PODN), and secreted protein acidic and rich in cysteine (SPARC) are commonly up-regulated among ileal strictures.

### PDGFRB Mediates Collagen Expression in Intestinal Fibroblasts

**Figure 2.**
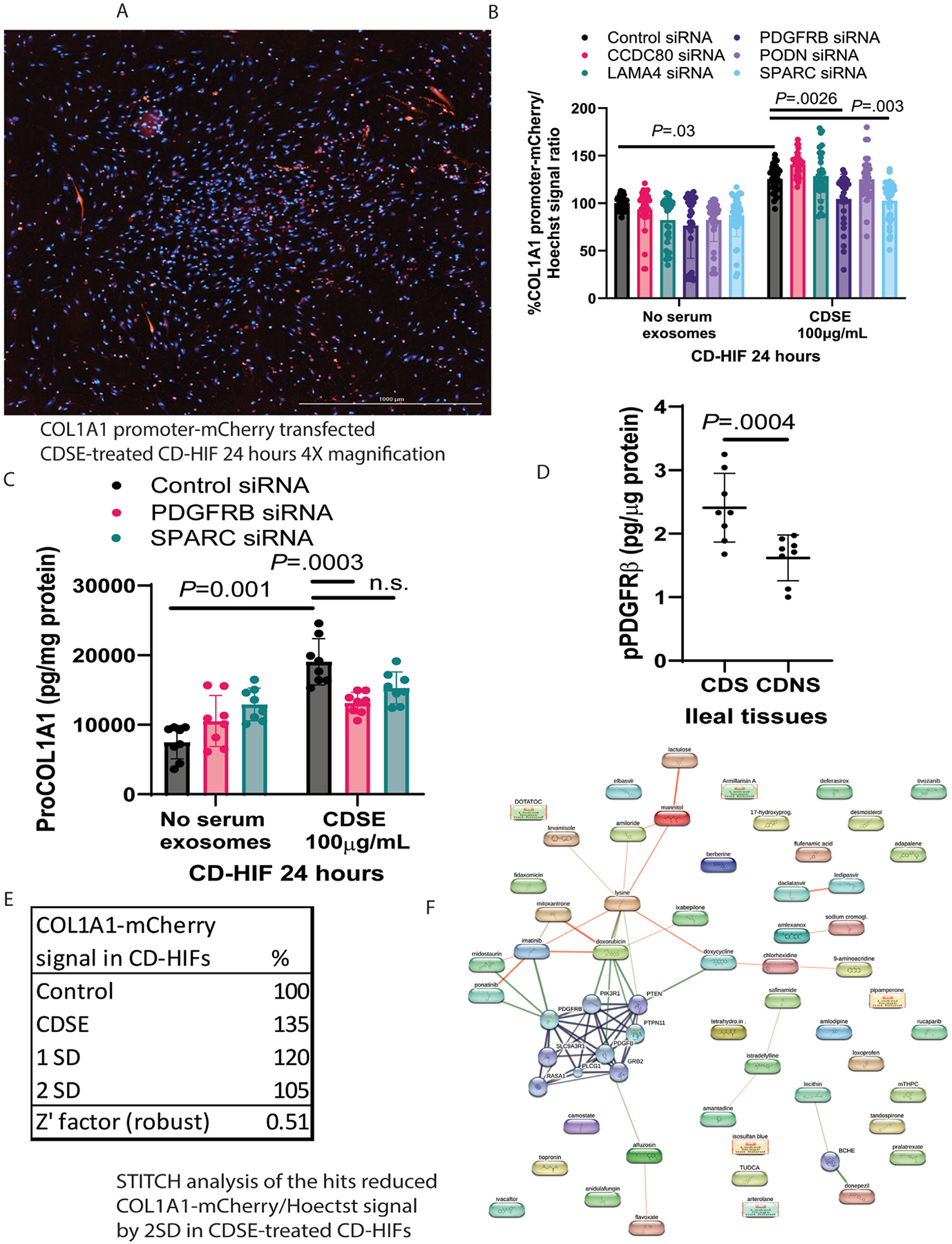
PDGFRB knockdown suppressed collagen expression in intestinal fibroblasts. (*A*) A sample image of COL1A1 promoter-mCherry transfected CDSE-treated CD-HIFs under 4× magnification. *Red* = COL1A1; *blue* = nuclei. (*B*) COL1A1 promoter activity assay. The CD-HIFs were transfected with 0.3 *μ*L/well siRNAs and 0.05 *μ*g/well COL1A1 promoter-mCherry construct via 5 *μ*L/well Opti-MEM, 0.3 *μ*L/well Lipofectamine 3000, and 100 *μ*L/well serum-free DMEM overnight, followed by 100 *μ*g/mL CDSE. After 24 hours, Hoechst nuclear stain was added. The percentage of the ratio of the COL1A1 red fluorescence over the Hoechst blue fluorescence indicated the relative collagen promoter activity. An increased ratio means increased fibrogenesis. Results were pooled from 4 experiments. Mean ± SD. One-way analysis of variance (ANOVA) was used. (*C*) The CD-HIFs were transfected with 0.3 *μ*L/well siRNAs and 0.05 *μ*g/well COL1A1 promoter-mCherry construct via 5 *μ*L/well Opti-MEM, 0.3 *μ*L/well Lipofectamine 3000, and 100 *μ*L/well serum-free DMEM overnight, followed by 100 μg/mL CDSE. After 24 hours, the protein lysates were used for ProCOL1A1 ELISA. Results were pooled from 4 experiments. Mean ± SD. One-way ANOVA was used. (*D*) Phosphorylated PDGFR*β* ELISA of frozen ileal explants from 4 CDS and 4 CDNS patients. Mean ± SD. A *t* test was used. (*E*) Ratios of the COL1A1 promoter-mCherry signal in red fluorescence and the Hoechst nuclear stain in blue fluorescence. Z’ factor >0.5 indicates valid assays. CDSE increased the COL1A1-mCherry signal, indicating increased collagen promoter activity in the CD-HIFs. (*F*) STITCH analysis of chemicals in the hit list. Two hits (Ponatinib and midostaurin) and a closely associated Imatinib are PDGFR*β* inhibitors. STITCH website: http://stitch.embl.de/cgi/input.pl?UserId=t4cULxleAjvc&sessionId=pym9G9ajKPkT.

CCDC80, LAMA4, PDGFRB, PODN, and SPARC are potential mediators of intestinal fibrosis due to common expression among ileal and colonic strictures (Figure 1F). Our group, therefore, established a new fluorescence-based imaging platform to assess COL1A1 promoter activity in CD-HIFs. COL1A1 is a pivotal readout for fibrogenesis in CD-HIFs.^13^ The COL1A1-mCherry transfected CD-HIFs produce red fluorescence when COL1A1 promoter activity occurs. As captured by the automated imager, profibrogenic CDSE activated COL1A1 promoter activity with increased mCherry signal intensity in the CD-HIFs (Figure 2A).

Transient knockdown of PDGFRB and SPARC, but not CCDC80, LAMA4, and PODN, inhibited the CDSE-mediated increase of COL1A1 promoter activity (Figure 2B). ELISA validation indicated that PDGFRB, but not SPARC, siRNA significantly reduced ProCOL1A1 protein expression in CDSE-treated CD-HIFs (Figure 2C), suggesting that PDGFRB regulated collagen expression in intestinal fibroblasts.

Ileal tissues from CDS patients had higher PDGFRb phosphorylation than those from nonstricturing Crohn’s disease (CDNS) patients (Figure 2D), suggesting an enhanced PDGFR*β* activity in intestinal strictures.

### PDGFRβ is an Actionable Molecular Target for Intestinal Fibrosis

To find actionable molecular targets of intestinal fibrosis, a drug screening assay with 2621 FDA-approved drugs using the fluorescent COL1A1 promoter workflow was performed. The assay was valid with a high Z’ factor (Figure 2E). CDSE was known to induce collagen expression in CD-HIFs.^11,13^ Fifty-one drugs (1.9%) inhibited COL1A1 promoter activity in the CDSE-treated CD-HIFs by 2 SDs (Supplementary Table 5). STITCH analysis of these hit compounds indicated that ponatinib and midostaurin are linked to PDGFRB (Figure 2F). They are PDGFR*β* inhibitors and therapeutic agents for leukemia.^19,20^ Thus, PDGFR*β* is an actionable molecular target in intestinal fibrosis.

### Fidaxomicin May Bind to PDGFRβ

Super-PRED, a machine-learning model, was used to predict drug-binding targets (Supplementary Table 6). Anticancer drugs midostaurin and ponatinib can target PDGFRA and PDGFRB.^21–23^ Due to their cytotoxic effects, they are unlikely to be used in clinics to treat CDS patients. Interestingly, fidaxomicin has a high predicted probability of binding to PDGFR (78%) and PDGFR alpha (92%; Supplementary Table 6). Fidaxomicin is an FDA-approved orally active antibiotic for treating CDI.^24^

**Figure 3.**
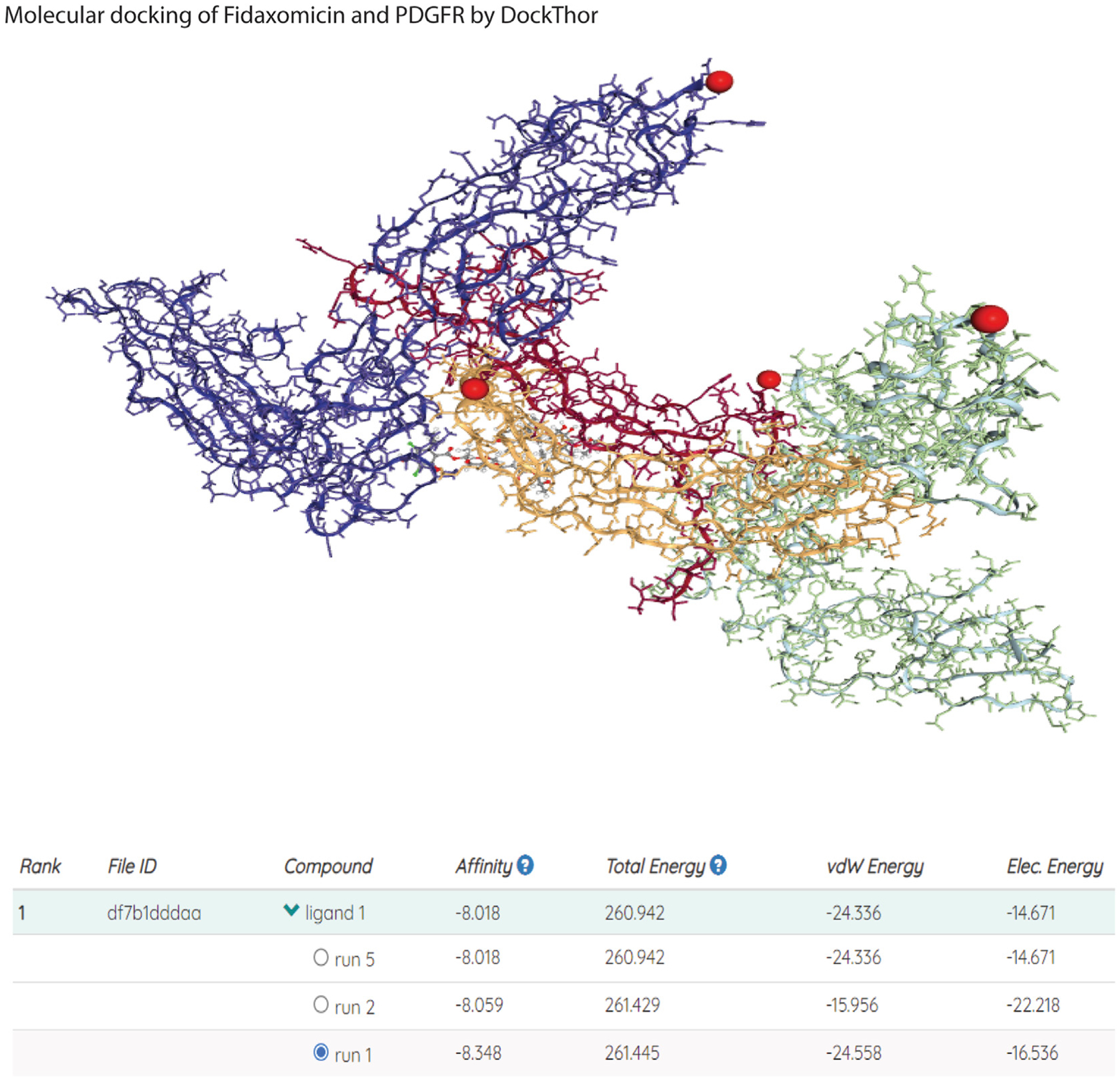
Molecular docking prediction analysis of fidaxomicin and PDGFR by DockThor. The PDGFR (3MJG) and fidaxomicin (FI8) structures were found in the RCSB PDB database. Four putative binding sites (indicated by *red dots*) are found. Affinity (kcal/mol) represents the binding affinity. The lowest affinity value (kcal/mol) represents the strongest binding between PDGFR and fidaxomicin. Research Collaboratory for Structural Bioinformatics Protein Data Bank (RCSB PDB) database website: https://www.rcsb.org/. DockThor website: https://dockthor.lncc.br/v2/#.

A molecular docking model (DockThor) predicted that fidaxomicin may bind to 4 sites on PDGFR (Figure 3). Three binding runs predicted strong binding between fidaxomicin and PDGFR with a low negative affinity value (from −8.018 to −8.348 kcal/mol). Another molecular docking model (CB-Dock2) also indicated that fidaxomicin may bind to 5 sites on PDGFR (Supplementary Figure 1A). The lowest negative Vina score (−8.5 kcal/mol) at the C4 site predicts strong binding between fidaxomicin and PDGFR (Supplementary Figure 1B). These results indicate the potential for fidaxomicin to inhibit PDGFRb and intestinal fibrosis.

### Fidaxomicin Inhibited Fibrogenesis Via PDGFRB Dephosphorylation and Down-Regulation in Intestinal Fibroblasts

**Figure 4.**
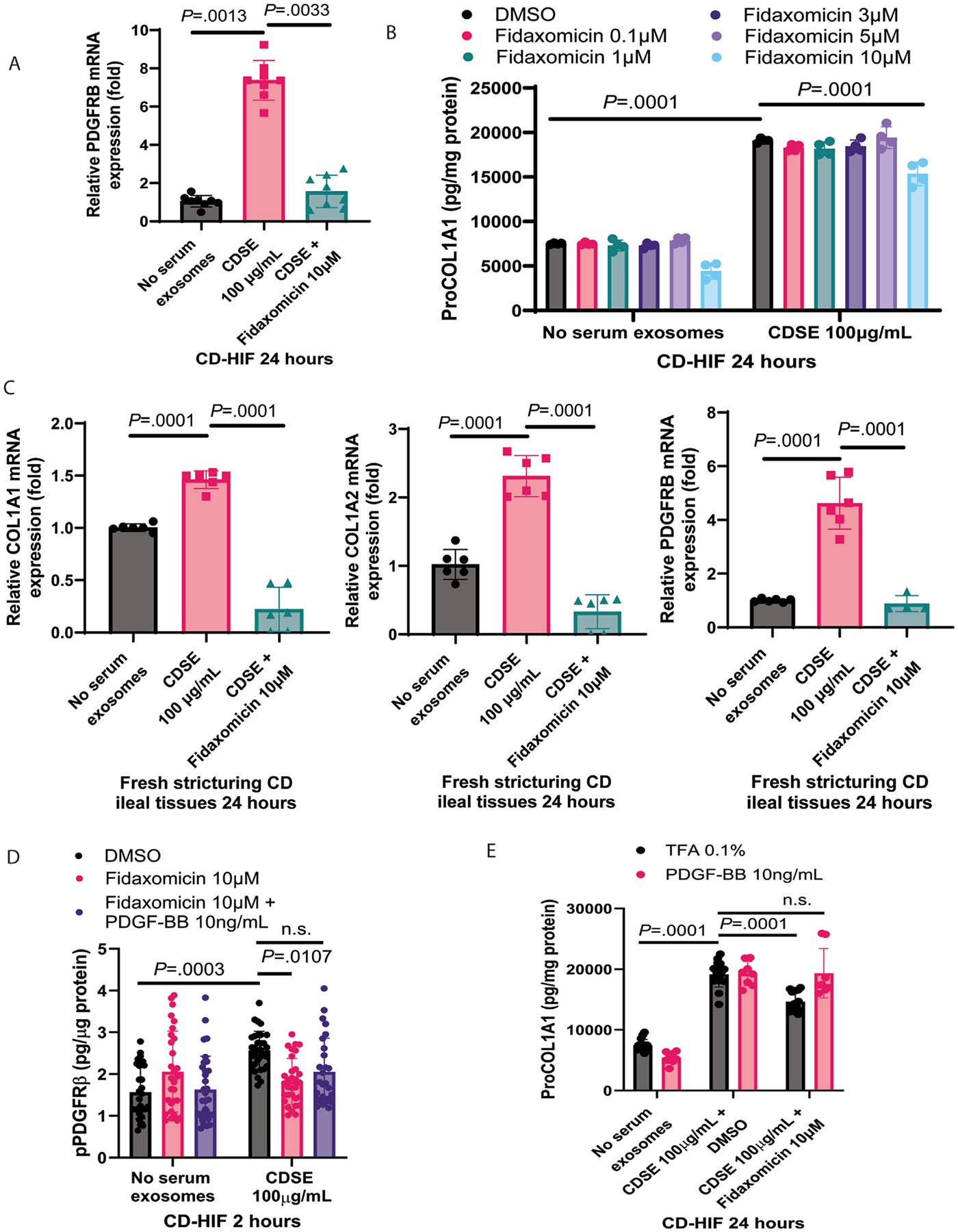
Fidaxomicin inhibited collagen expression in intestinal fibroblasts via PDGFR*β* inhibition. (*A*) Real-time RT-PCR. CD-HIFs were pretreated with CDSE for 30 minutes, followed by fidaxomicin for 24 hours. Relative PDGFRB mRNA expression was normalized to the 18S ribosomal RNA (rRNA) gene. Fidaxomicin at 10 *μ*M decreased CDSE-induced PDGFRB mRNA expression in CD-HIFs. Mean ± SD. One-way ANOVA was used. Results were pooled from 4 experiments. (*B*) CD-HIFs were pretreated with CDSE for 30 minutes, followed by various concentrations of fidaxomicin for 24 hours. ProCOL1A1 protein expression was determined using ELISA. Fidaxomicin at 10 *μ*M significantly reduced ProCOL1A1 expression in CDSE-treated CD-HIFs. Mean ± SD. One-way ANOVA was used. Results were pooled from 4 experiments. (*C*) Real-time RT-PCR. Fresh stricturing CD patient-derived ileal explants were exposed to CDSE for 30 minutes, followed by DMSO or fidaxomicin for 24 hours. Relative PDGFRB, COL1A1, and COL1A2 mRNA expression were normalized to the 18S rRNA gene. Fidaxomicin at 10 *μ*M significantly reduced ProCOL1A1 expression in CDSE-treated fresh CDS ileal tissues. n = 4 CDS patients. Mean ± SD. One-way ANOVA was used. (*D*) Phosphorylated PDGFRb ELISA. CD-HIFs were pretreated with CDSE for 30 minutes, followed by fidaxomicin and PDGF-BB for 2 hours. Fidaxomicin inhibited CDSE-induced PDGFR*β* phosphorylation, mildly attenuated by PDGF-BB. Mean ± SD. One-way ANOVA was used. Results were pooled from 4 experiments. (*E*) ProCOL1A1 ELISA. CD-HIFs were transfected with COL1A1-mCherry construct. The CD-HIFs were pretreated with CDSE and PDGF-BB for 30 minutes, followed by fidaxomicin for 24 hours. ProCOL1A1 quantification was determined using ELISA. Fidaxomicin reduced ProCOL1A1 protein expression, which was abolished by PDGF-BB. Mean ± SD. One-way ANOVA was used. Results were pooled from 4 experiments.

Real-time reverse transcription-polymerase chain reaction (RT-PCR) indicated that 24-hour CDSE treatment increased PDGFRB mRNA expression in CD-HIFs, which was reduced by 10 *μ*M fidaxomicin (Figure 4A). Consistent with our previous study,^13^ CDSE increased collagen ProCOL1A1 protein expression in CD-HIF (Figure 4B). Ten *μ*M fidaxomicin was optimal for inhibiting ProCOL1A1 protein expression in CDSE-treated CD-HIFs (Figure 4B). As shown using whole-transcriptome RNA sequencing, fidaxomicin significantly reduced collagen and PDGFRB mRNA expression in CDSE-treated CD-HIFs (Supplementary Table 7). Similarly, 10 *μ*M fidaxomicin abolished the CDSE-mediated increase in COL1A1, COL1A2, and PDGFRB mRNA expression in CDS patient-derived fresh ileal explants (Figure 4C).

Two-hour CDSE treatment increased PDGFR*β* phosphorylation in the CD-HIFs, which was dephosphorylated by fidaxomicin (Figure 4D). Platelet-derived growth factor-BB (PDGF-BB) is a preferred ligand for the PDGFRb*β*.^25^ Ten ng/mL PDGF-BB partially abolished the fidaxomicin-mediated inhibition of PDGFRb phosphorylation and ProCOL1A1 protein expression in CDSE-treated CD-HIFs (Figure 4D and E). These results suggested that the anti-fibrogenic effect of fidaxomicin is dependent on the dual inhibition of PDGFR*β* expression and phosphorylation.

### Fidaxomicin Mediated GSK3β Dephosphorylation in Intestinal Fibroblasts

**Figure 5.**
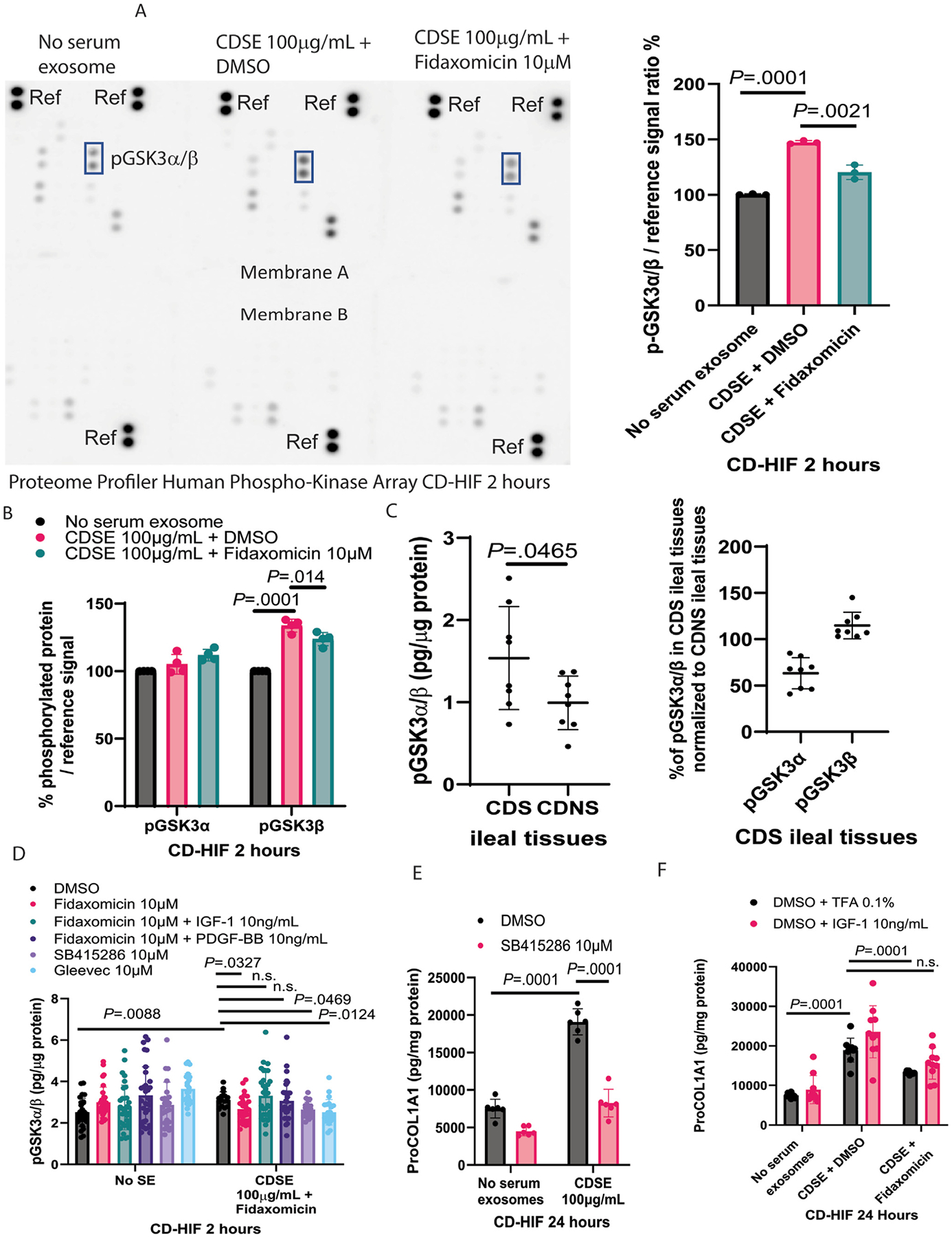
Fidaxomicin inhibited collagen expression in intestinal fibroblasts via GSK3*β* inhibition. (*A*) Phospho-kinase array. CD-HIFs were pretreated with CDSE for 30 minutes, followed by fidaxomicin for 2 hours. The 300 μg protein/group lysates were loaded into the Proteome Profiler Human Phospho-Kinase Array Kit (ARY003C, R&D Systems). A Bio-Rad ChemiDoc Imaging system detected protein signals on protein arrays. Relative GSK3ɑ/*β* phosphorylation normalized to the reference signals. Protein signals on the protein arrays were quantified by Bio-Rad Image Lab software. Fidaxomicin inhibited CDSE-induced GSK3ɑ/*β* phosphorylation in CD-HIFs. Mean ± SD. One-way ANOVA was used. Results were pooled from 3 experiments. (*B*) Phosphorylated GSK3ɑ and GSK3*β* ELISA. CD-HIFs were pretreated with CDSE for 30 minutes, followed by fidaxomicin for 2 hours. Fidaxomicin inhibited CDSE-induced GSK3*β* phosphorylation in CD-HIFs. CDSE and fidaxomicin did not affect GSK3ɑ phosphorylation. Mean ± SD. One-way ANOVA was used. Results were pooled from 4 experiments. (*C*) Phosphorylated GSK3ɑ/*β* ELISA, phosphorylated GSK3ɑ ELISA, and phosphorylated GSK3*β* ELISA. (Left) Phosphorylated GSK3ɑ/*β* ELISA of frozen ileal tissues from 4 CDS and 4 CDNS patients. (Right) The levels of pGSK3ɑ and pGSK3*β* in 4 CDS ileal explants were normalized to the 4 CDNS ileal explants. CDS ileal tissues had weaker GSK3ɑ and stronger GSK3*β* phosphorylation than CDNS ileal tissues. Mean ± SD. A *t* test was used. (*D*) Phosphorylated GSK3ɑ/*β* ELISA. CD-HIFs were pretreated with CDSE for 30 minutes, followed by fidaxomicin, PDGF-BB (PDGFR*β* activator), IGF-1 (GSK3 phosphorylation activator), SB415286 (GSK3 inhibitor), and Gleevec (PDGFR inhibitor) for 2 hours. Fidaxomicin, SB415286, and Gleevec inhibited GSK3ɑ/*β* phosphorylation. PDGF-BB and IGF-1 counteracted fidaxomicin’s inhibition of GSK3ɑ/*β* phosphorylation. Mean ± SD. One-way ANOVA was used. Results were pooled from 4 experiments. (*E*) ProCOL1A1 ELISA. CD-HIFs were pretreated with 100 *μ*g/mL CDSE for 30 minutes, followed by GSK3 inhibitor SB415286 for 24 hours. ProCOL1A1 protein expression was determined using ELISA. Mean ± SD. One-way ANOVA was used. Results were pooled from 3 experiments. SB415286 inhibited collagen expression. (*F*) ProCOL1A1 ELISA. CD-HIFs were pretreated with 100 *μ*g/mL CDSE for 30 minutes, followed by 10 *μ*M fidaxomicin and IGF-1 (GSK3 phosphorylation activator) for 24 hours. ProCOL1A1 protein expression was determined using ELISA. Mean ± SD. One-way ANOVA was used. IGF-1 abolished fidaxomicin’s inhibition of collagen expression. Results were pooled from 4 experiments.

PDGFR*β* can cross-talk with other intracellular signaling molecules,^26^ which may mediate fidaxomicin’s effects. Protein arrays showed that CDSE increased GSK3ɑ/*β* phosphorylation in CD-HIFs, which was diminished using fidaxomicin (Figure 5A). ELISA experiments showed that CDSE increased GSK3*β* but not GSK3ɑ phosphorylation, which was reduced by fidaxomicin (Figure 5B). Ileal tissues from CDS patients have higher phosphorylated GSK3ɑ/*β*, specifically higher GSK3*β* and lower GSK3ɑ levels than those from nonstricturing CD patients (Figure 5C). Phosphorylated GSK3 is inactive, while dephosphorylated GSK3 is active.^27^ Thus, fidaxomicin-mediated GSK3*β* dephosphorylation keeps GSK3*β* active.

### Fidaxomicin Mediated PDGFRβ-Dependent GSK3b Dephosphorylation in Intestinal Fibroblasts

Consistent with another report,^28^ GSK3 phosphorylation inhibitor SB415286 inhibited GSK3ɑ/*β* phosphorylation in the CDSE-treated CD-HIFs (Figure 5D). On the other hand, insulin-like growth factor 1 (IGF-1) can deactivate GSK3ɑ/*β* by inducing GSK3ɑ/*β* phosphorylation.^27^ GSK3ɑ/*β* phosphorylation depends on PDGFR*β* because PDGF-BB can induce GSK3ɑ/*β* phosphorylation.^29^ Inhibition of PDGFR*β* by Gleevec (also known as Imatinib) also dephosphorylated GSK3ɑ/*β* (Figure 5D). As PDGF-BB and IGF-1 abolished the fidaxomicin-mediated GSK3ɑ/*β* dephosphorylation in CDSE-treated CD-HIFs (Figure 5D), fidaxomicin dephosphorylated GSK3*β* via PDGFR*β* inhibition.

### Fidaxomicin Inhibited Fibrogenesis Via GSK3β Inhibition in Intestinal Fibroblasts

Like fidaxomicin, the GSK3 inhibitor SB415286 abolished the CDSE-mediated collagen expression in CD-HIFs (Figure 5E). Cotreatment with IGF-1 diminished the anti-fibrogenic effect of fidaxomicin in CDSE-treated CD-HIFs (Figure 5F), suggesting that fidaxomicin inhibited fibrogenesis in CD-HIFs via GSK3*β* inhibition.

### Fidaxomicin Did Not Affect Fibroblast Migration and Epithelial-Mesenchymal Transition

Transforming growth factor beta 1 (TGF-*β*1) is a profibrogenic factor. TGF-b1 can induce the migration of intestinal fibroblasts and epithelial-mesenchymal transition–like collagen transcriptional activity in HPECs,^13,30^ but these phenomena were unaffected by fidaxomicin (Supplementary Figures 2A and B). On the other hand, epithelial-mesenchymal transition–like collagen promoter activity was also unaffected by siRNA knockdown of stricture-related targets (CCDC80, LAMA4, PDGFRB, PODN, and SPARC; Supplementary Figure 2C), suggesting that PDGFRB mediates fibrogenic activity in intestinal fibroblasts but not intestinal epithelial cells.

### Fidaxomicin Exerted Anti-Inflammatory Effects in PBMCs

Lipopolysaccharide (LPS) is a bacterial endotoxin and a potent proinflammatory stimulus. Fidaxomicin significantly reduced LPS-mediated interleukin 8 and tumor necrosis factor alpha (TNFɑ) secretion in CD patient-derived PBMCs (Supplementary Figure 2D), suggesting that fidaxomicin modulated proinflammatory cytokine secretion in immune cells.

CDSE, not LPS, mildly stimulated secretion of profibrogenic TGF-*β*1, but not proinflammatory interleukin 8 and TNFɑ, secretion in CDS-PBMCs (Supplementary Figure 2D).

Fidaxomicin did not affect CDSE-mediated TGF-*β*1 secretion in CDS-PBMCs (Supplementary Figure 2D).

### Oral Fidaxomicin Treatment Ameliorated Ileal Fibrosis in CD-Like SAMP1/YitFc Mice

**Figure 6.**
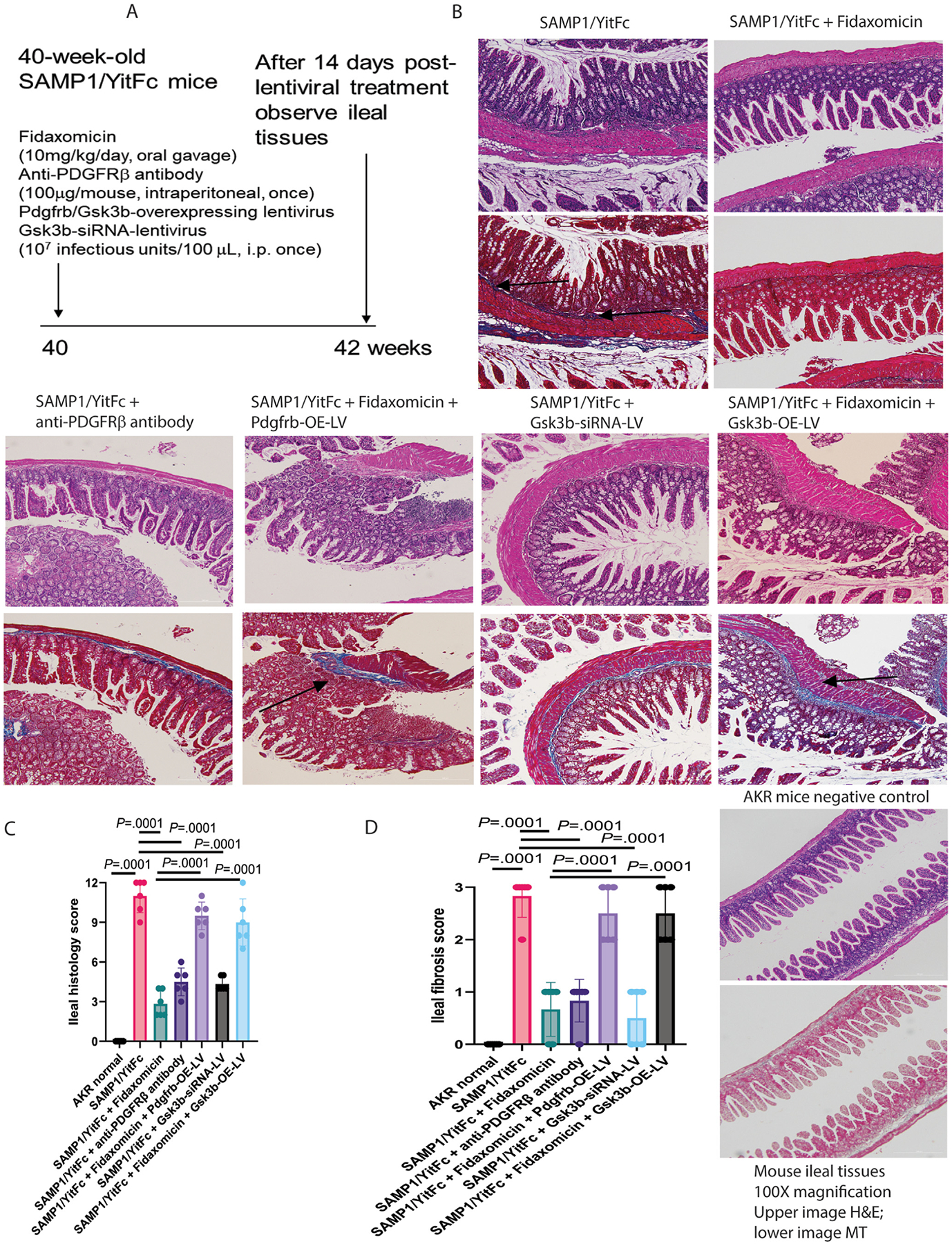
Oral fidaxomicin treatment ameliorated ileal fibrosis in SAMP1/YitFc mice. (*A*) Experimental plan of the ileal fibrosis study in SAMP1/YitFc mice. Interventions were started at 40 weeks of age when the SAMP1/YitFc mice had established ileal fibrosis. Specimens were collected at 42 weeks of age. (*B*) H&E and MT staining images of the ileal tissues. The blue Masson’s trichrome (MT) staining indicated collagen deposition, as indicated by *arrows*. SAMP1/YitFc mice had ileal fibrosis. AKR mice are negative controls. Fidaxomicin, anti-PDGFR*β* antibody, and Gsk3b-siRNA lentivirus treatment reduced ileal fibrosis. Pdgfrb- and Gsk3b-overexpressing lentiviruses abolished the anti-fibrogenic effect of fidaxomicin. 300-*μ*m *scale bars* are displayed at the lower right corners. (*C* and *D*) Ileal histology score and fibrosis score. Mean ± SD. One-way ANOVA was used. n = 6 mice per group. Two rounds of experiments.

Forty-week-old SAMP1/YitFc mice with spontaneous CD-like ileitis and ileal fibrosis were used to determine the mechanisms of action of oral fidaxomicin treatment (Figure 6A).^17,31^ Consistent with our previous study,^13^ the 42-week-old SAMP1/YitFc mice had ileal mucosal injuries and fibrosis compared with the normal-appearing ileal structure in their control AKR mice (Figure 6B). These general histologic observations were quantified by histology and fibrosis scores. Oral fidaxomicin treatment restored the normal ileal structures, with a significant reduction in ileal histology and fibrosis scores in the SAMP1/YitFc mice (Figures 6B–D).

Ileal histology and fibrosis scores and real-time RT-PCR data were included to calculate the overall disease activity (ODA).^13^ Fidaxomicin reduced the ileal mRNA expression of fibrogenic (Col1a2, Col3a1, Zeb1, Vim, and Acta2) and inflammatory (Tnf and Emr1) genes in the SAMP1/YitFc mice, leading to lowered ODA (Supplementary Table 8).

### PDGFRβ and GSK3β Mediated Ileal Fibrosis in SAMP1/YitFc Mice

**Figure 7.**
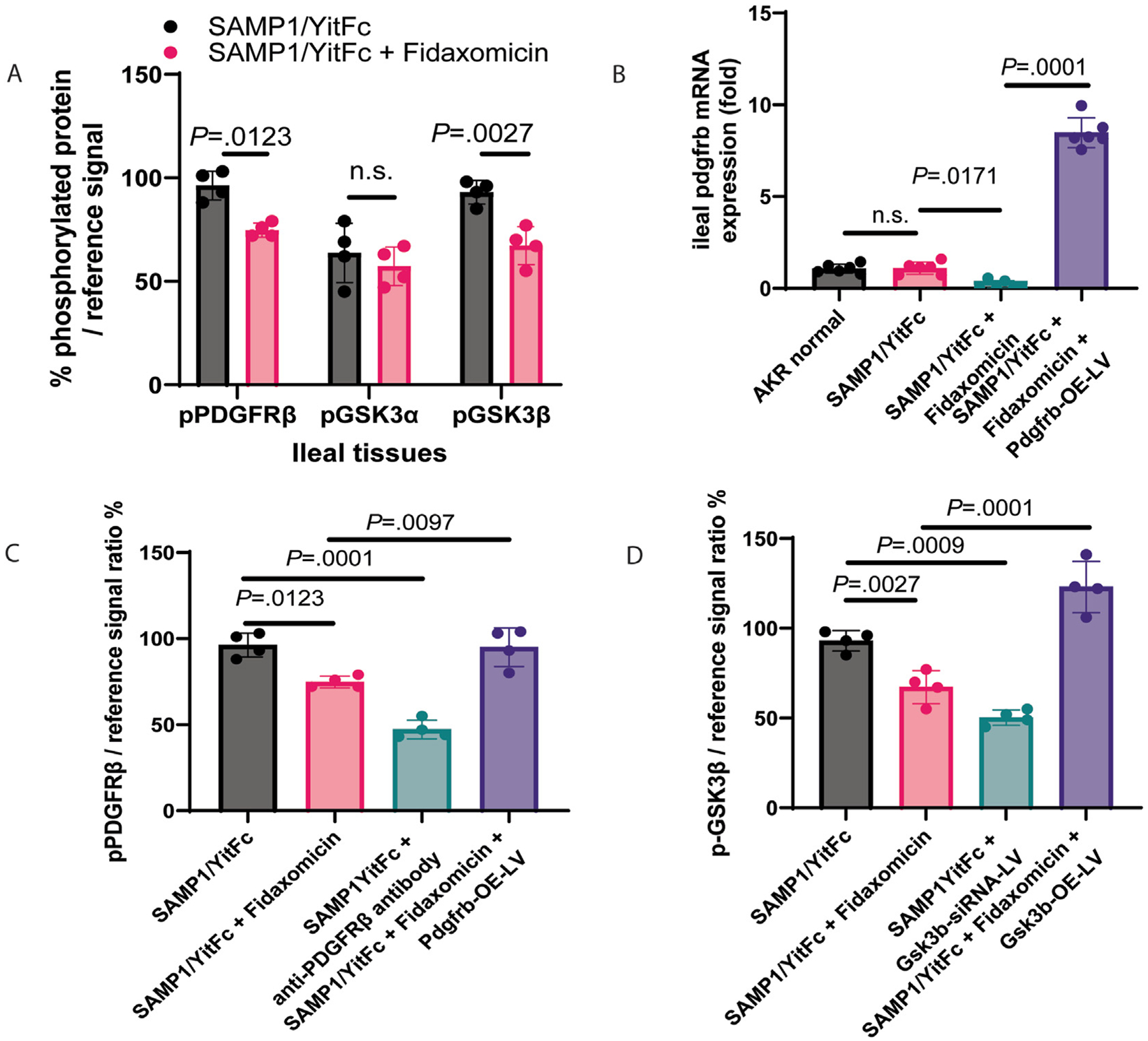
Oral fidaxomicin treatment inhibited ileal fibrosis in SAMP1/YitFc mice by inhibiting Pdgfrb and GSK3b. (*A*) Phosphorylated PDGFR*β*, GSK3ɑ, and GSK3*β* ELISA. Fidaxomicin reduced ileal pPDGFR*β* and GSK3*β* phosphorylation in SAMP1/YitFc mice. Mean ± SD. *t* tests were used. N = 4 mice per group. (*B*) Real-time RT-PCR. Relative Pdgfrb mRNA expression was normalized to the Gapdh gene. Fidaxomicin reduced ileal Pdgfrb mRNA expression in SAMP1/YitFc mice, which was abolished by Pdgfrb-overexpressing lentivirus. Mean ± SD. One-way ANOVA was used. N = 6 mice per group. (*C*) Phosphorylated PDGFR*β* ELISA. Fidaxomicin reduced ileal PDGFR*β* phosphorylation in SAMP1/YitFc mice, which was abolished by Pdgfrb-overexpressing lentivirus. Mean ± SD. One-way ANOVA was used. N = 4 mice per group. (*D*) Phosphorylated GSK3b ELISA. Fidaxomicin reduced ileal GSK3*β* phosphorylation in SAMP1/YitFc mice, which was abolished by Gsk3b-overexpressing lentivirus. Mean ± SD. One-way ANOVA was used. N = 4 mice per group. All samples were assayed in 1 round of the experiment.

As in CD-HIFs (Figures 4A, 4D, and 5B), oral fidaxomicin treatment reduced ileal Pdgfrb mRNA expression and PDGFR*β* and GSK3*β* but not GSK3ɑ phosphorylation in SAMP1/YitFc mice (Figures 7A–D). As PDGFR*β* inhibitors (Midostaurin and Ponatinib) and GSK3*β* inhibitors (Cromolyn and SB415286) inhibited collagen expression in CDSE-treated CD-HIFs (Supplementary Table 5, Figure 5E), inhibition of these targets with anti-PDGFR*β* antibody and Gsk3b-siRNA lentivirus also ameliorated ileal fibrosis and reduced ODA in SAMP1/YitFc mice (Figures 6B–D, Supplementary Table 8). Notably, injections of anti-PDGFR*β* antibody and Gsk3b-siRNA lentivirus inhibited ileal PDGFR*β* and GSK3*β* phosphorylation, respectively (Figures 7C and D). Thus, both PDGFR*β* and GSK3*β* mediated ileal fibrosis in SAMP1/YitFc mice.

### Oral Fidaxomicin Treatment Ameliorated Ileitis and Ileal Fibrosis in SAMP1/YitFc Mice Via Ileal PDGFRβ and GSK3β Inhibition

The effects of fidaxomicin on ileitis and ileal fibrosis were abolished by lentiviral Pdgfrb and Gsk3b overexpression (Figure 6B). The fidaxomicin-mediated reduction of ileal histology and fibrosis scores and ODA was partially abolished by lentiviral Pdgfrb and Gsk3b overexpression (Figures 6C and D, Supplementary Table 8).

### Oral Fidaxomicin Inhibited Ileal Pdgfrb mRNA Expression and PDGFRβ and GSK3β Phosphorylation in SAMP1/YitFc Mice

Fibrotic SAMP1/YitFc and nonfibrotic AKR mice had similar ileal Pdgfrb mRNA expression (Figure 7B). Fidaxomicin’s suppression of ileal Pdgfrb mRNA expression, PDGFR*β* phosphorylation, and GSK3*β* phosphorylation in the SAMP1/YitFc mice was abolished by lentiviral Pdgfrb and Gsk3b overexpression, respectively (Figures 7B–D). Therefore, the antifibrogenic effect of fidaxomicin was mediated by PDGFR*β* down-regulation and dephosphorylation and GSK3*β* dephosphorylation.

### Oral Fidaxomicin Treatment Did Not Affect Ileal Microbiota in SAMP1/YitFc Mice

Fidaxomicin was desirable for treating CDI because of its microflora-sparing properties.^32^ Similarly, shotgun metagenomic sequencing showed that oral fidaxomicin treatment did not affect ileal alpha diversity, beta diversity, and relative abundance of dominant bacterial species in the SAMP1/YitFc mice (Supplementary Figure 3). For comparison, 20-week oral antibiotic treatment failed to reduce the spontaneous ileitis and ileal fibrosis in SAMP1/YitFc mice, as reflected by histology and fibrosis score (Supplementary Figures 4A–C). This antibiotic mixture was used to suppress intestinal microbiota and facilitate CDI in mice.^33^ Therefore, such a chronic gut microbiota suppression cannot affect ileal fibrosis in SAMP1/YitFc mice.

## Discussion

This study used advanced approaches to identify actionable molecular targets in intestinal fibrosis. Unlike whole-transcriptome RNA sequencing, spatial RNA sequencing explored the local gene signatures in CDS patients’ most fibrotic regions compared with adjacent, less fibrotic regions in ileal tissues. By comparing our datasets and others,^7,11^ commonly overexpressed genes in ileal fibrosis were found. The functional study and high-throughput screening showed that PDGFRB is the actionable gene mediating fibrogenesis in intestinal fibroblasts (Figure 2). FDA-approved fidaxomicin-mediated inhibition of collagen expression and PDGFR*β* activity and expression in CDS patient-derived fibroblasts and human and mouse intestinal tissues is clinically relevant for the precision treatment of intestinal strictures among CD patients.

The FDA approved fidaxomicin for treating CDI colitis in 2011.^24^ Fidaxomicin’s superiority over other PDGFR*β* inhibitors is its minimal absorption into the systemic circulation.^34^ Fidaxomicin mainly remains in the gut and is eventually excreted via feces. Thus, there is little concern about fidaxomicin affecting PDGFR*β* activity in other organs, and fidaxomicin is associated with few mild adverse reactions.^35^

The PDGF system is actively involved in multiple kinds of organ fibrosis.^36^ PDGFR*β* is a molecular target for lung fibrosis because anti-PDGFR*β*, but not anti-PDGFRɑ, antibodies inhibit bleomycin-induced pulmonary fibrosis in mice.^37^ Renal mesenchymal PDGFRb activation induced kidney failure and fibrosis in mice.^38^ These findings provide a strong rationale for studying PDGFR*β* as a target in intestinal fibrosis. However, imatinib/Gleevec, an FDA-approved antileukemia drug and PDGFR*β* inhibitor, is known to cause multiple adverse drug reactions, including thrombocytopenia.^39^ Systemic inhibition of PDGFR*β* with cytotoxic anti-PDGFR*β* drugs may be risky. Instead, the minimal absorption of fidaxomicin into circulation can maintain the high exposure of the stricturing intestine to the fidaxomicin and reduce risks to other organs.^40^

Another benefit of fidaxomicin is the preservation of intestinal microbiota in patients.^32^ Like our SAMP1/YitFc model of spontaneous ileal fibrosis (Supplementary Figure 4), the same antibiotic treatment also could not reduce colonic fibrosis in trinitrobenzene sulfonic acid–treated mice.^41^ These 2 mouse models suggested that microbial suppression is unnecessary to inhibit intestinal fibrosis. As oral fidaxomicin treatment did not affect ileal microbiota (Supplementary Figure 3), the antimicrobial property of fidaxomicin was not associated with its anti-fibrogenic effect in the fibrotic SAMP1/YitFc mice.

GSK3ɑ/*β* is involved in fibrosis in the heart, lung, liver, and kidney.^42^ GSK3 inhibitor treatment could inhibit renal fibrosis in mice.^43^ Lithium chloride is the only approved GSK3 inhibitor for treating epilepsy and bipolar disorder.^44^ The utility of GSK3 inhibitors in treating fibrotic diseases was not tested in clinical trials.

Mild weight loss was observed among fidaxomicin-, anti-PDGFR*β*-neutralizing antibody-, and Gsk3b-siRNA-lentivirus-treated SAMP1/YitFc mice (Supplementary Table 9). Such a mild body weight loss was not concerning because the decrease was only up to 9%. Fidaxomicin-mediated weight loss was restored by lentiviral Pdgfrb and Gsk3b overexpression (Supplementary Table 9). PDGFR*β* and GSK3 may regulate body weight, as PDGFR*β* and GSK3 deficiencies reduce high-fat diet-induced obesity in mice.^45,46^ Fidaxomicin is minimally absorbed into the circulation, so it is speculated that its inhibition of intestinal PDGFR*β* and GSK3*β* may regulate body weight indirectly.

In addition to its antifibrogenic effects, fidaxomicin’s anti-inflammatory effects in CDS-PBMCs and ileal tissues of SAMP1/YitFc mice may be relevant to CD (Supplementary Figure 2D, Supplementary Table 8). Similarly, fidaxomicin exerts anti-inflammatory and cytoprotective effects against *C difficile* toxins in human colonic explants.^47^ Fidaxomicin can inhibit *C difficile* toxin-mediated cytokine release in macrophages and apoptosis in colonic epithelial cells via nuclear factor (NF)-κB inhibition.^47^ Fidaxomicin may interact with NF-κB to modulate inflammation because the Super-PRED predicts the NF-κB p105 subunit as a target of fidaxomicin with a 97.16% probability (Supplementary Table 10). NF-κB is a crucial transcriptional factor for mediating inflammation and TNFɑ expression in PBMCs.^48,49^ The anti-inflammatory effect of fidaxomicin may help treat CD because NF-κB activation positively correlates with histologic scores in CD patients.^50^ Although the role of anti-inflammatory medications in intestinal stricture development among CD patients is controversial,^1,7^ the dual anti-inflammatory and anti-fibrogenic effects of fidaxomicin may confer synergistic protection to CDS patients.

Although fidaxomicin has not been approved to treat CD patients, a phase 3b/4 trial (PROFILE) found fidaxomicin to be safe and well-tolerated in patients with active inflammatory bowel disease and CDI.^34^ Fidaxomicin may also be repurposed to treat other diseases, as it improves survival in mice with leukemia by affecting leukemia-associated signaling molecules.^51^

Serum samples from healthy donors, CDS patients, and CDNS patients were assayed with proteomics. There were no significant changes in circulating PDGF-related ligands and receptors and SPARC among CDS patients (Supplementary Table 11). These factors in circulation are unlikely to affect intestinal fibrosis.

A limitation of this study is the small sample size of human-derived ileal tissues due to their limited availability. Fresh ileal tissues and CD-HIFs were treated with fidaxomicin up to 24 hours (Figure 4). Due to the high cost and limited availability of SAMP1/YitFc mice, the SAMP1/YitFc mice were treated with fidaxomicin for 2 weeks (Figure 6). The study of the long-term effects of fidaxomicin treatment was unavailable. Therefore, preclinical models of human cells and tissues and mice cannot fully replicate CD strictures. The applicability of fidaxomicin in treating CD strictures still needs to be evaluated in clinical trials.

Our 10X Genomics Visium spatial RNA sequencing discovered PDGFRB as a signature gene in fibrotic strictures from adult CDS patients (Figure 1), consistent with fibrotic strictures from pediatric CDS patients.^7^ Two groups performed 10X Genomics Chromium single-cell RNA sequencing of intestinal tissues from CDS patients and used PDGFR as a fibroblast marker.^52,53^ However, these 2 studies show different findings, probably due to patients’ different characteristics.^52,53^ The blood and surgically resected ileal tissues were collected from CD patients with fibrotic ileal strictures but varying degrees of inflammation, such as differences in C-reactive protein and fecal calprotectin (Supplementary Tables 1 and 2), which may have introduced heterogeneity into the data analysis.

Fidaxomicin’s inhibition on ProCOL1A1 protein expression in CD-HIFs (Figure 4B) was weaker compared with fidaxomicin’s inhibition on COL1A1 and COL1A2 mRNA expression in fresh CDS ileal tissues (Figure 4C). They are different assays using different materials. Several factors, including post-translational modification and protein turn-over, may affect protein expression. Additionally, technical variability, such as the starting material and experimental factors, makes the quantitative comparison between mRNA and protein expression difficult.

In summary, PDGFR*β* and GSK3*β* are actionable targets in intestinal fibrosis. Fidaxomicin inhibits PDGFR*β* phosphorylation and expression and GSK3*β* phosphorylation in intestinal fibroblasts. Due to its minimal gut absorption and microbial interference characteristics, FDA-approved fidaxomicin has excellent potential to provide a safe and effective therapeutic strategy for treating intestinal fibrosis. Our workflow reveals an accelerated method for discovering actionable targets and finding new antifibrogenic agents.

## Supplementary Material

supplementary material

1

Note: To access the supplementary material accompanying this article, visit the online version of Gastroenterology at www.gastrojournal.org, and at https://doi.org/10.1053/j.gastro.2025.04.028.

## Data Availability

All data, analytical methods, and study materials will be available to other researchers. Please contact Dr Koon.

## WHAT YOU NEED TO KNOW

### BACKGROUND AND CONTEXT

Fifty percent of Crohn’s disease patients develop intestinal strictures. Discovering new targets and agents is imperative because no medication is available to inhibit intestinal fibrosis.

### NEW FINDINGS

Platelet-derived growth factor receptor beta and glycogen synthase kinase-3 beta are crucial mediators of collagen expression in intestinal fibroblasts. Fidaxomicin mediated an antifibrogenic effect by inhibiting these mediators.

### LIMITATIONS

The number of samples of human-derived ileal tissues is small. Preclinical models of human cells and tissues and mice cannot fully replicate Crohn’s disease strictures.

### CLINICAL RESEARCH RELEVANCE

Fidaxomicin is an approved drug that is not absorbed into circulation. Fidaxomicin remains in the gut, increasing the strictured intestinal exposure to the drug.

### BASIC RESEARCH RELEVANCE

Platelet-derived growth factor receptor beta and glycogen synthase kinase-3 beta are new molecular targets of intestinal fibrosis, which allow further discovery of their pathogenic mechanisms.

## Abbreviations used in this paper:

ANOVA: analysis of variance
CCDC80: coiled-coil domain-containing 80
CD: Crohn’s disease
CDI: *Clostridioides difficile* infection
CDNS: nonstricturing Crohn’s disease
CDS: stricturing Crohn’s disease
CDSE: stricturing Crohn’s disease patient-derived serum exosomes
COL1A2: collagen 1A2
DMEM: Dulbecco’s Modified Eagle Medium
ECM: extracellular matrix
ELISA: enzyme-linked immunosorbent assay
FDA: Food and Drug Administration
GSK3ɑ/*β*: glycogen synthase kinase-3 alpha/beta
HIFs: human intestinal fibroblasts
HPECs: primary human intestinal epithelial cells
IGF-1: insulin-like growth factor 1
LAMA4: laminin subunit alpha 4
LPS: lipopolysaccharide
mRNA: messenger RNA
NF: nuclear factor
ODA: overall disease activity
PBMC: peripheral blood mononuclear cell
PDGF-BB: platelet-derived growth factor-BB
PDGFR*β*: platelet-derived growth factor receptor beta
PODN: podocan
ProCOL1A1: pro-collagen I alpha 1
SD: standard deviation
siRNA: small interfering RNA
SPARC: secreted protein acidic and rich in cysteine
TGF: transforming growth factor
TNF: tumor necrosis factor
UCLA: University of California Los Angeles
