# Supplementary material for: Fidaxomicin Reduces Collagen Expression in Intestinal Fibroblasts Via Platelet-Derived Growth Factor Receptor Beta and Glycogen Synthase Kinase-3 Beta Inhibition
Source: Gastroenterology. Author manuscript; Available in PMC 2026 Jan 21. (PMC12822550; doi:10.1053/j.gastro.2025.04.028)
Supplement: supplementary material [file NIHMS2137854-supplement-supplementary_material.pdf]

Supplementary Table 1

ID	age	F=0 M=1 sex	mg/L CRP	mm/h ESR	ug/g calprotectin	doctor's note	stricture site	WBC	Hemoglobin	hematocrit	BMI	no=0 yes=1 current smoking	no-0 yes=1 current alcohol	IBD related Medications	no=0 yes=1 steroid	no=0 yes=1 stricture	IBD related surgery	HBI Score
WH009A	51	1	13.2	>100		small bowel thickening, focal obstructuve process and possible partial obstruction.	ileum	13.3	7.8	26.2	16.0	0	0	Prednisone	1	1	short bowel surgery	
WH020A	57	0	3.4	38	421	recurrent fibrous stricturing crohn's	ileum	8.5	11.4	34.3	40.6	1	1	none	1	1	none	7 or 8
WH024	44	1	<0.3	14		stricturing small bowel crohn's, mid to distal small bowel, small bowel resection	ileum	7.8	15.0	44.4	23.4	0	1	adalimumab	0	1	2 bowel resections and strictureplasty	1
WH028A	36	0	0.9	16*		CD of distal & terminal ileum with strictures	ileum	5.6	13.2	39.4	22.5	0	0	mesalamine [Pentasa]	0	1	none	0
WH032	25	1	26.3	35	>2500	stricturing Crohn's disease diagnosed in 2012 and he has current suprapubic and LLQ abdominal pain, which is unusual for him. He usually has RUQ abdominal pain	ileum	22.9	11.9	37.0	17.7	0	0	adalimumab	0	1	none	
WH042	36	0	1	20		stricturing CD	ileum	11.6	11.5	35.8	26.6	1	0	none	0	1	none	15
WH062	26	1	12.9	46		stricturing CD	ileum	10.5	9.6	30.3	16.3	0	0	none	0	1	none	8

Supplementary Table 2

CD01/CD04
Collection date: 10/7/2021
CDS ileum
Montreal classification: A3 L1 B2
BS267215
Age: 41-45 yo
Sex: Male
Diagnosis: Crohn’s small bowel stricture
Location: Ileum
Medications:
budesonide 3 mg 24 hr capsule, take 9 mg by mouth
dicyclomine 10 mg capsule, take 20 my by mouth
multiple vitamin tablet, take 1 tablet by mouth
ondansetron ODT (ZOFRAN ODT) 4 mg disintegrating tablet, take 8 mg by mouth.
Stelara 90mg/mL injection, inject 1 syringe under the skin every 8 weeks.
Melatonin PO, take by mouth.

CD02/03
Collection date: 3/7/2022
CDS ileum
Montreal classification: A2 L1 B2
BS274692
Age: 26-30 yo
Sex: Female
Disease type: Crohn's disease of small intestine with intestinal obstruction
Disease location: ileum
“26-30 y.o. year-old female with PMH Crohn's (diagnosed 2017) with recurrent episodes of SBO presenting for consultation of surgical management of stricturing disease. Patient reports she was diagnosed with Crohn's in 2017 and was on Humira from 2019 - 2020. She felt it was not effective and decided to stop taking in August 2020. She was not on any medication for 1-1.5yrs. She has had numerous episodes of hospital admission for SBO since coming off Humira and additional episodes for which she does not seek medical attention. During these episodes she will sometimes have feculent vomit. She reports episodes last ~2 days and steroids while admitted helped resolve these episodes. Accordingly, she was started on daily PO prednisone by PCP, dose titrated up to 40mg daily current dose.”

CD05A
Collection date: 11/10/2022
CDS ileum
Montreal classification: A3 L1 B2
Sex: Female
Age: 51-55yo
Dg: Crohn’s disease of ileum with stricture
Location: Ileum
Patient was placed on Humira in 6/2022. Currently on Remicade induction.

CD06A
Collection date: 12/21/2022
Normal ileum
Montreal classification: Not applicable
Sex: Female
Age: 51-55
Disease: Neuroendocrine cancer of small intestine
tissue location: mid ileal small bowel

CD07
Collection date: 1/12/2023
CDS ileum
Montreal classification: A2 L1 B2
Stricture/fibrotic portion of terminal ileum
Sex: Male
Age: 26-30
Disease: Crohn’s disease
Tissue location: terminal ileum
Medication: Excerpt from patient’s chart “Since initial consultation in 11/2021, the patient has established care and initiated infliximab (he did not tolerate methotrexate) to which he has responded but he remains prednisone dependent and whenever he tries to taper below 15 mg he develops increasing symptoms.”

Supplementary Table 3

CD-HIFs and HPECs				
Patient	1	2	3	4
Age	41	45	56	58
Gender	Male	Female	Male	Female
Disease	CD stricture	CD stricture	CD stricture	CD stricture
Disease location	ileum	ileum	ileum	ileum

Supplementary Table 4

CD02 cluster 4	CD02 cluster 8	CD04 cluster 2	CD05A cluster 1	CD05A cluster 2
A2M	A2M	ACTA2	ASM	ADAM28
ACTA2	ACTA2	ADAMTS4	ABCC9	ADAMDEC1
ACTN4	ACTN4	ADGRL1	ACKR1	ADM
ADAMTS1	ADAM33	AEBP1	ACTA2	AKIRIN2
ADGRE5	ADAMTS1	ARMC9	ACTG2	AKNA
ADIRF	ADGRE5	ATOH8	ADAMTS9	ALOX5
AEBP1	ADIRF	CCN2	ADGRA2	ANKZF1
ATF3	AEBP1	CHRM2	AEBP1	ANP32E
BGN	ATF3	COL12A1	AHNAK2	APOE
C1orf198	BGN	COLEC12	AKAP6	ARRB2
C3	C1orf198	CRYBG3	ALDH1A3	CASP8
CCDC80	C3	CSRP2	AMOTL1	CCDC71L
CCL19	CCDC80	CTXN1	ARMC9	CCN1
CCN1	CCN1	DGKB	BHLHE40	CD163
CCN2	CCN2	DSTN	C14orf132	CFD
CD79A	CFH	EFNA5	C3	CFH
CFH	CITED2	EPHA4	CALD1	CHST11
CITED2	CLIC4	EPPK1	CAP2	CNTRL
COL1A1	COL1A1	FLNC	CAV1	COL23A1
COL1A2	COL1A2	GALR2	CCDC69	COL3A1
COL3A1	COL3A1	GFRA1	CCN1	CORO1A
CPE	CPE	GNG11	CITED4	CRACR2A
CTSK	CTSK	GPR161	CLDN5	CRELD2
CXCR4	DUSP1	GREM2	CLIC4	CXCl14
CYBA	EGR1	HIF3A	CLU	CXCR4
DDIT4	ELN	HMCN2	CNN1	CYTIP
DUSP1	EMILIN1	HPGD	COL12A1	DDTL
EGR1	ESYT2	HPSE2	COL15A1	DEFA5
ELN	FMOD	HTR2B	COL3A1	DEFA6
FABP4	FOS	IGFBP3	CRIP2	EGFL7
FMOD	FOSB	IL1RL1	CSPG4	ELN
FOS	FRZB	JAM2	CSRP1	ERN1
FOSB	GAS1	KIAA1522	CST3	FAM118A
FRZB	GREM1	LAG3	DCN	FBN1
GAS1	IGF1	LONRF2	DEPP1	FGD2
GREM1	IGFBP7	MAB21L2	DES	FOSB
HERPUD1	INMT	MAOB	DMPK	GDF5
IGF1	LASP1	MN1	DPT	GNL3
IGFBP7	LBH	MT-ND3	EGR1	GPX3
IGHA1	LTBP1	MYH11	FBLN1	HBA2
IGHG1	LUM	NPTX1	FBN1	HCLS1
IGHG2	MFGE8	PAWR	FOXF1	HELLS
IGHG3	MMP2	PCDH7	FSTL1	IER5
IGHG4	MRC2	PDE5A	FXYD1	IFI16
IGHJ2	MYH10	PI15	GADD45B	IGHA1
IGHM	MYLK	PITPNM2	GFRA1	IGKC
IGKC	NBL1	PTGIS	GPX3	IKZF1
IGKV4-1	NNMT	PTGS2	GRASP	IL1R1
IGLC1	PCDH7	PTN	GREM1	IL7R
IGLV3-1	PCOLCE	RGMA	GUCY1A1	IRAK3
INMT	PDGFRB	RUFY4	HAND2	ITGA6
ISG20	PDLIM5	SCARA3	HSPB6	ITLN2
JCHAIN	PLA2G2A	SGCA	HSPB7	JAML
LBH	PLTP	SHROOM3	HSPB8	JCHAIN
LTBP1	PODN	SORBS2	IDH2	KLF2
LUM	PTGDS	SPON1	IFITM3	KLHL6
LYZ	PTGER3	TCIM	IGHG1	LAP3
MFGE8	PTGIS	THBS1	IL1R1	LIFR
MMP2	RAMP1	TSC22D1	ITIH5	LMNB2
MRC2	SERPINE1	WFDC2	JAM3	LSP1
MYH10	SERPINF1	WNT9A	JUNB	LYZ
MZB1	SERPING1		KCTD12	MMP19
NNMT	SFRP2		KLF2	MMRN1
PCOLCE	SLIT3		LDHB	MRFAP1L1
PDGFRB	SMOC2		LIFR	MT-ND5
PDLIM5	SOD3		LYVE1	MT-ND6
PECAM1	SPARC		MAB21L1	ODF3B
PLA2G2A	SRPX		MAP1B	OXNAD1
PLTP	TGM2		MAP3K20	PFKBP3
PODN	THBS2		MGP	PGGHG
POU2AF1	THY1		MYL9	PHLDA1
PTGDS	TIMP2		MYLK	PIK3R3
PTGER3	TSPAN7		NNMT	PLA2G2A
PTGIS	VCAN		NOTCH3	PLTP
RNASET2			NRXN3	PLXND1
S100A4			OGN	PPFIBP1
SCD			PDGFRA	PRDM1
SERPINF1			PDGFRB	PTGDS
SERPING1			PDLIM3	QTRT1
SFRP1			PECAM1	RAP2B
SFRP2			PHLDA1	RASA2
SLIT3			PI15	RASGRP2
SMAP2			PLIN4	REG3A
SMOC2			PODN	S100A4
SOD3			PRELP	SFMBT2
SPARC			PRIMA1	SH3BP5
SRPX			PRSS23	SIGIRR
SSR4			PRUNE2	SLA
STK17B			PTGIS	SLBP
TENT5C			RAMP1	SMAP2
TGM2			RASSF3	STAB1
THBS2			RERG	STK4
THEMIS2			RGMA	TAF11L6
THY1			RGS5	TBX1
TIMP2			RNF150	TCF21
TSPAN7			S100A4	TMEM63A
VCAN			SERPING1	TMSB4X
VWF			SH3BP5	TNXB
XBP1			SLC25A4	TRAC
			SOC3	TSC22D3
			SORBS2	TXNDC5
			SPARC	USP17L15
			SVIL	ZAP70
			SYNM	ZNF394
			TGFBR1	ZNF549
			THBS1	ZNF550
			TNXB	
			TPM2	
			TSC22D1	
			TSPAN18	
			VIP	
			VWA1	
			VWF	
			WFDC2	

Supplementary Table 5

Inhibited COL1A1-mCherry in CDSE-treated CD-HIF

FDA panel

9-Aminoacridine	isosulfan blue
Alfuzosin	Istradefylline
Amantadine	Ixabepilone
amiloride (hydrochloride)	Lactitol (monohydrate)
Amlexanox	lactulose
Amlodipine	Lecithin
Amlodipine	Ledipasvir
Amlodipine (maleate)	levamlodipine besylate
anidulafungin	L-lysine hydrochloride
Armillarisin A	Loxoprofen
Arterolane	Metergoline
Belotecan (hydrochloride)	Midostaurin
Berberine sulfate	mitoxantrone (dihydrochloride)
Camostat (mesylate)	Pipamperone
Cromolyn (sodium)	Ponatinib
Daclatasvir	pralatrexate
Deferasirox Fe3+ chelate	Protamine sulfate
Desmosterol	Ripretinib
Doxorubicin	Rucaparib (phosphate)
Doxycycline	Tandospirone (citrate)
D-sorbitol	Tauroursodeoxycholate
Edotreotide	temoporfin
Elbasvir	Tetrahydrobiopterin
Fidaxomicin	Tetramisole
Flavoxate (hydrochloride)	Tivozanib hydrochloride sulfate
Guanethidine (sulfate)	

Supplementary Table 6

	Probability %				
**Super-PRED target prediction**	**PDGFR**	**PDGFRA**	**PDGFRB**	**average**	**Targets**
9-Aminoacridine					Bacterial; HIV
Adapalene	67			67	Apoptosis; autophagy; RAR/RXR
Alfuzosin	56	63		59	Adrenergic receptor
Amantadine	52	52		52	Apoptosis; Bcl-2 family; CDk; influenza virus; orthopoxvirus; SARS-CoV
amiloride (hydrochloride)	53			53	apoptosis; sodium channel; TRP channel
Amlexanox					IKK
Amlodipine		69		69	calcium channel
anidulafungin	88			88	antibiotic; fungal
Armillarisin A		58		58	interleukin related
Arterolane	51	75		63	Parasite
Berberine sulfate		51		51	Autophagy; bacterial; parasite; reactive oxygen species; Topoisomerase
Camostat (mesylate)					SARS-CoV; Ser/Thr Protease
Chlorhexidine	73			73	antibiotic; bacterial
cromolyn (sodium)		57		57	clacium channnel; GSK-3
Daclatasvir	59			59	HCV
Deferasirox Fe3+ chelate					Bacterial; Ferroptosis
Desmosterol	59	62		60	Endogenous metabolite
Donepezil hydrochloride		61		61	Cholinesterase (CHE)
Doxorubicin	63	78		70	ADC cytotoxin; AMPK; Antibiotic; apoptosis; autophagy; bacterial; HBV; HIV;mitophagy; Topoisomerase
Doxycycline	70	69		69	Antibiotic; bacterial; MMP; parasite
D-sorbitol	58	54		56	bacterial; endogenous metabolite
Edotreotide					others
Elbasvir					HCV
**Fidaxomicin**	78	92		85	bacterial, orally active antibiotic
Flavoxate (hydrochloride)		66		66	calcium channel; MAChR; Phosphodiesterase (PDE)
flufenamic acid		73		73	AMPK; calcium channel; chloride channnel; COX; parasite; potassium channel
Hydroxyprogesterone caproate		69		69	others
isosulfan blue		62		62	others
Istradefylline	87			87	Adenosine receptor
Ivacaftor		89		89	autophagy; CFTR
Ixabepilone	63			63	apoptosis; bacterial; microtubule/tubulin
lactulose	63	55		59	bacterial; endogenous metabolite
Lecithin	69	55		62	Endogenous metabolite
Ledipasvir	69	54		62	HCV; SARS-CoV
Lenoalidomide					apoptosis; ligands for E3 ligase; molecular glues
levamlodipine besylate		53		53	calcium channel
L-lysine hydrochloride		58		58	antifolate; apoptosis; autophagy
Loxoprofen	52	51		51	COX
Mavacamten					Myosin
**Midostaurin**		100	75	88	Apoptosis; c-Kit; NO synthase; PKC; VEGFR; **PDGFR**
Mitoxantrone (dihydrochloride)	52			52	apoptosis; endogenous metabolite; orthopoxvirus; PKC; Topoisomerase
Pipamperone	83	92		87	5-HT receptor; dopamine receptor
**Ponatinib**	73	100	100	91	Autophagy; Bcr-Abl; FGFR; **PDGFR**; Src; VEGFR
pralatrexate	52			52	antifolate; apoptosis
Protamine sulfate					Thrombin
**Ripretinib**					Apoptosis; c-Kit; FLT3; **PDGFR**; VEGFR
Rucaparib					PARP
Rucaparib phosphate		57		57	GNSR
safinamide		78		78	monoamine oxidase
Tandospirone (citrate)					5-HT receptor
Tauroursodeoxycholate	51	63		57	Apoptosis; caspase; Endogenous metabolite; ERK
temoporfin					others
Tetrahydrobiopterin					Endogenous metabolite; NO synthase
Tetramisole					antibiotic; parasite; phosphatase
tiopronin	55	60		57	others
Tivozanib hydrochloride sulfate					VEGFR

Supplementary Table 7

CDSE vs. Control

Upregulated

ADGRE2, ADRA2C, AFDN-DT, AMDHD1, ANKRD18B, ANLN, ASF1B, BHLHA15, BLM, BMS1P15, C5orf34, CCNE2, CDCA2, CENPE, CENPF, CENPK, CEP55, CFAP46, CHORDC1P1, CIT, CKAP2L, CYCSP55, CYP4A22-AS1, DCAF4L1, DDIAS, DEPDC1, DLGAP5, DLSTP1, DTL, DUSP6, DYNLT5, E2F2, EEF1A1P10, EPHA1-AS1, EPIST, ERCC6L, FABP5P7, FAM111B, FGFR3, FOLR3, GFAP, GOLGA8J, GPR3, GPR78, H3-5, HJURP, HNRNPA1P35, HSP90AA4P, IQGAP3, ISM1, KIF11, KIF14, KIF18A, KIF23, KIF24, KIFC1, KLC2-AS1, KNL1, LAT, LINC01431, LINC02056, LINP1, LMNB1, MAD2L1, MCCD1P2, MDGA2, MIR1244-3, MIR3661, MKI67, MTFP1, MYCL, MYO3B, NCAPG, NCAPH, NECTIN3-AS1, NOP2, NR4A2, NUF2, ODAD2, OR2A1, ORC6, PHOSPHO1, PKMYT1, POLQ, POTEE, PRMT5-DT, PROSER2-AS1, PRSS27, RADIL, RFC3, RN7SL336P, RPL29P12, RPL37AP1, RPL39P15, RPS6P25, RPSAP18, RPSAP61, RTN4RL1, S100A1, SCN5A, SEMA6A-AS1, SHCBP1, SKA3, SLC10A5, SLC25A5-AS1, SMG7-AS1, SNAP25, SNAP91, SNORD3A, SPATA17, SPTBN5, TAF12-DT, TBX3-AS1, TM4SF19-AS1, TMEM158, TMX2P1, TNFSF15, TOB2P1, TOP2A, TP73, TRBV12-4, TTK, UHRF1, VNN2, VPS9D1-AS1, XIRP2, YTHDF3-DT, ZNF367, ZNF485, ZNF625, ZRANB2-AS1

Downregulated

AATK, ACSS2, ALPK3, ANGPTL1, APOBEC3D, APOBEC3G, APOL1, APOL6, ARHGAP27, ATG12P1, BATF2, BIRC3, BST2, CASP12, CCL5, CDH3, CEACAM1, CEBPA, CIRBP-AS1, CRYM-AS1, CTAGE4, CXADR, CXCL10, DAZAP2P1, DDX60, DDX60L, DHX58, DNAAF8, DTX3L, EEF1E1P1, EIF2AK2, EPSTI1, FBXO32, FLNC-AS1, GBP1, GEMIN7-AS1, GJD3, GRIP1, H4C12, HOXD1, IFI27, IFI35, IFI44, IFIH1, IFITM1, IL18BP, IL4I1, INHBB, IRF1-AS1, IRF7, ISG20, LBHD1, LGALS9, LHFPL3-AS2, LINC01311, LINC02055, LINC02392, LINC02795, LINC02823, LRRC4, LRRN3, MAN2A1-DT, MIR616, MPHOSPH10P3, MYG1, NCF2, NHLH1, NLRC5, NRN1, NRXN1, NT5C3AP1, OASL, PALM3, PAQR6, PARP12, PARP14, PARP9, PIK3AP1, PKD1L2, PLEKHA4, PLSCR1, PNPT1, POU5F1, PPM1K, PRKCZ-AS1, PTOV1-AS2, RASEF, REPIN1-AS1, RGS7, RIMS4, RNF207-AS1, RNU6-9, RRAGD, RTP4, RWDD3-DT, SAMD9, SAMD9L, SCARF1, SCARNA7, SCN1A-AS1, SERF1A, SLC22A1, SP110, STAT1, STYK1, SUGCT-AS1, SULT1C2, TAP1, THEMIS2, TM4SF1-AS1, TMC1, TNFSF10, TNFSF13B, TRIM21, TRIM22, TRIM25, TTC9, TYMP, UBA7, UBBP4, WAPL-DT, XAF1, ZNF524, ZNFX1

CDSE + Fidaxomicin versus CDSE + DMSO

Downregulated

A1BG-AS1, A4GALT, AAMDC, ABCA6, ABCA8, ABCC6, ABCD2, ABTB1, ACADS, ACAT1, ACBD4, ACCS, ACER3, ACP3, ACSF2, ACSS1, ACVRL1, ADAM19, ADAM33, ADAMTS2, ADAMTS8, ADAMTSL4, ADARB1, ADD3, ADGRA2, ADGRL3, ADH1B, ADRA1B, ADSS1, AGRN, AGT, AKR7A2, ALAD, ALDH1A1, ALDH1A3-AS1, ALDH3A2, ALDH3B1, ALDH7A1, ALG6, AMOT, AMPH, ANG, ANGPT1, ANGPTL2, ANLN, ANO4, ANTXR1, ANXA13, ANXA4, AP4M1, APCDD1, APOBEC3B, APOBEC3C, ARHGAP11A, ARHGAP20, ARHGAP23, ARHGAP28, ARHGAP5-AS1, ARHGAP6, ARHGEF17, ARHGEF25, ARID5B, ARMC9, ARNT2, ARPIN, ARRB1, ARRDC1, ASF1B, ASIC1, ASL, ASPA, ASPM, ATOH8, ATP6V0D1-DT, ATP8B4, ATRIP, AUH, AVIL, B3GALT2, B4GAT1, BAALC-AS1, BACE1, BAHCC1, BAIAP2-DT, BAZ2B-AS1, BBS2, BCAT2, BCL9L, BDKRB2, BEX3, BICC1, BIRC5, BLM, BLNK, BMF, BMP4, BMP5, BMPER, BNC2-AS1, BRAT1, BRCA1, BST1, BTG1, BTG2, BUB1, BUB1B, C10orf55, C14orf132, C14orf93, C16orf89, C1GALT1C1L, C1orf112, C1orf21, C1QTNF1, C1QTNF1-AS1, C1QTNF2, C1QTNF6, C1RL, C21orf58, C22orf39, C2orf15, C2orf88, C3orf62, C4orf3, C4orf33, C4orf46

C6, CABLES1, CACNA2D3, CACNB2, CACNB3, CALD1, CALHM2, CARD16, CARHSP1, CASP1, CAT, CAV1, CAVIN1, CAVIN2, CBFA2T3, CBLN3, CBR4, CBX2, CBX7, CC2D2A, CCBE1, CCDC102B, CCDC28B, CCDC69, CCDC8, CCDC89, CCL11, CCL13, CCL2, CCL7, CCN4, CCNA2, CCNB2, CCND2, CD200, CD248, CD27-AS1, CD302, CD36, CD59, CDC20P1, CDC25B, CDC25C, CDCA3, CDCA5, CDCA8, CDH11, CDH6, CDK1, CDK15, CDK2, CDKN1C, CEBPD, CENPA, CENPE, CENPF, CENPH, CENPI, CENPK, CENPP, CEP128, CEP55, CEP68, CFTR, CHEK2, CHL1-AS2, CHN1, CHRM2, CHST14, CHST7, CIBAR1, CIBAR1-DT, CIBAR1P1, CILK1, CKAP2L, CKS1B, CLDN11, CLEC12A, CLN8-AS1, CNP, CNPY4, CNTNAP3, COL13A1, COL14A1, COL1A2, COL25A1, COL3A1, COL5A1, COL5A2, COL8A1, COLEC10, COQ2, CPZ, CREB3L1, CRISPLD1, CRISPLD2, CROCC, CSF1, CTDSP1, CTDSP2, CTDSPL, CTHRC1, CTSC, CTSO, CXCL12, CYB5A, CYB5RL, CYGB, CYP1B1, CYP24A1, CYRIA, CZIB, DAAM2, DAB1, DCAF11, DCN, DDAH2, DDB2, DEAF1, DENND2A, DENND2B, DEPDC1, DEPTOR, DGUOK-AS1, DHFR

DHRS1, DIAPH3, DLG3, DLGAP5, DNAH5, DNAI3, DNAJC22, DNAJC9, DNALI1, DPM3, DPYD, DPYSL2, DPYSL3, DSCC1, DSN1, DTX4, DUS4L-BCAP29, DUT, E2F8, EDNRB, EEF2K, EFCC1, EFNA1, EFNB3, EHD2, EHD3, EID1, EMILIN1, EMILIN3, EMP2, EMSY-DT, ENDOV, ENO3, ENOX1, EPB41L2, EPHA7, EPS8L2, EPSTI1, ERBB2, ERCC6L, ERVMER34-1, EVL, EXO1, EXTL1, EYA1, F8, F8A1, FAM107A, FAM111B, FAM117B, FAM13C, FAM151B-DT, FAM20C, FAM210B, FAM43A, FAM83D, FANCA, FANCC, FANCD2, FANCI, FAXDC2, FBLN1, FBLN5, FBXO43, FBXW4, FDXR, FENDRR, FGF14, FGF7, FGFR4, FGGY, FHL1, FIBIN, FKBP5, FLOT2, FMO2, FMO3, FN3KRP, FOSL2, FOSL2-AS1, FOXF1, FOXF2, FOXM1, FOXO1, FSTL1, FTOP1, FUT11, FUT8, FUT8-AS1, FZD1, FZD2, FZD4, FZD7, GAL3ST4, GALNT11, GALNT15, GALNT2, GALNT5, GAS1, GAS1RR, GAS6-AS1, GASAL1, GASK1B, GCNT1, GDF5, GDF6, GGCX, GINS1, GLI2, GLIPR2, GLIS1, GLT8D2, GLUL, GMDS, GNG11, GNG7, GPD1L, GPR162, GPRC5B, GRAMD1C, GRAMD4, GRIA1, GRK5, GSDMD, GSEC, GSN, GTF2IRD1P1, GTF2IRD2, GTF2IRD2B, GTSE1, GUCY1A1, GUCY1B1, GYG2

H1-10, H1-10-AS1, H1-2, H2AC6, H2AZ2, H2BC11, H2BC19P, H3-3A, H3P6, H6PD, HAAO, HACD4, HAND2, HAND2-AS1, HAS2, HASPIN, HAUS5, HCFC1R1, HDAC5, HELLS, HEXIM1, HGF, HHLA3, HIRIP3, HJURP, HMGB1, HMGB1P6, HMGB3, HMGCLL1, HMGN2, HMGN2P5, HMGN3, HMMR, HNMT, HOPX, HOXB7, HOXC8, HPS6, HR, HRCT1, HS6ST1, HSD11B1, HSD17B11, HSPA2, HTR2B, HTRA1, HYAL2, IAH1, IBA57, ICMT, ID1, IDH1-AS1, IER5L, IFITM10, IFT140, IGF2-AS, IGIP, IKBKB-DT, IL16, IL1RN, IMMP2L, INKA2, INMT, INO80D-AS1, INPP5D, IP6K3, IQCC, IQGAP3, IRAG1, IRF2BPL, IRS1, ITGA3, ITGB8, ITGB8-AS1, ITGBL1, JADE1, JUP, KANK2, KCND1, KCND3, KCNH5, KCNH7, KCNK12, KCNN3, KIAA2012, KIAA2012-AS1, KIF11, KIF15, KIF18A, KIF18B, KIF20A, KIF20B, KIF22, KIF23, KIF24, KIF26B, KIF2C, KIF4A, KIFC1, KLF10, KLF9, KLRF1, KNL1, KRT18P19, KSR1, LAMC1, LBH, LDB1, LHFPL1, LHPP, LIG1, LINC00242, LINC00475, LINC00519, LINC00578, LINC00840, LINC00842, LINC00933, LINC00997, LINC01011, LINC01082, LINC01116, LINC01127, LINC01237, LINC01238, LINC01447, LINC01475, LINC01619, LINC01778, LINC01963, LINC02147, LINC02482, LINC02511

LINC02580, LINC02606, LINC02615, LINC02762, LINC02709, LINC02803, LINC02884, LMNA, LMNB1, LMO3, LMOD1, LNX1, LPAR1, LPAR6, LPCAT3, LPP-AS2, LRIG3, LRRC17, LRRC32, LRRC57, LRRK1, LRRK2-DT, LRRN4CL, LRRTM2, LSAMP, LTBP4, LUM, LYNX1, LYRM9, MAB21L2, MAD2L1, MAF, MAMLD1, MAN1B1, MAN1C1, MAOA, MAP3K12, MAP3K2-DT, MAP4K3-DT, MAPK10, MAPK7, MARVELD1, MASP1, MBL1P, MBLAC1, MBNL1-AS1, MCM4, MCM5, MCM7, MEIS1, MEIS1-AS2, MELK, METTL7A, MFAP4, MFSD6, MGAT4B, MIB2, MICALL2, MIDN, MINDY1, MIR1915HG, MIR210HG, MIR4500HG, MIR924HG, MKI67, MKS1, MLLT6, MMD, MMP11, MN1, MOB3B, MOSPD3, MRC2, MRGPRF, MRGPRF-AS1, MROH6, MTARC1, MTARC2, MTCL1, MTURN, MUC1, MUSK, MVP, MXD4, MXRA5, MYH13, MYLK, MYOCD, N4BP3, NAA80, NAALADL1, NACAD, NAPEPLD, NAT14, NAV1, NBL1, NBPF2P, NBPF3, NCAPD2, NCAPG, NCAPG2, NCAPH, NDC80, NDN, NDST1, NEGR1, NEIL1, NEIL3, NEK2, NEK6, NEK8, NEXN, NFATC4, NFIA, NFIX, NFYB, NIBAN2, NIFK-AS1, NIPSNAP2, NKD2, NKX2-3, NLGN1, NLGN2, NMNAT2, NNMT, NOTUM, NR2F1, NR2F2, NR2F2-AS1, NREP, NRK

NUDCD2, NUDT3, NUF2, NUMBL, NXN, NYNRIN, OCA2, OCEL1, OIP5, OLFM1, OLFML1, OLFML2A, OLFML2B, OLFML3, OLR1, OMG, OPN3, ORAI3, OSBPL5, OSBPL7, OXCT1, OXER1, P2RY14, PALM, PAMR1, PAPLN, PAQR4, PAQR7, PARPBP, PBK, PBLD, PBX1, PCDH18, PCDH7, PCNA, PCYOX1, PDCD4, PDE1A, PDE5A, PDE7B, PDGFD, PDGFRA, PDGFRB, PDPN, PDZRN3, PDZRN4, PEG10, PGM5P3-AS1, PGM5P4, PGM5P4-AS1, PHF19, PHLDB1, PHYHD1, PIDD1, PIEZO2, PIK3R3, PIMREG, PINK1, PITX1, PITX1-AS1, PKD1L1-AS1, PKDCC, PLAT, PLB1, PLCB3, PLCD3, PLCE1, PLD2, PLEC, PLEKHA6, PLEKHG2, PLEKHO2, PLK1, PLK4, PLPP1, PLPP3, PLXNB1, PLXNB2, PMP22, PNMA8B, PODN, PODNL1, POLA1, POLE, POLE2, POLH, POLR3GL, PPFIA4, PPL, PPM1M, PPP1R14A, PPP1R1C, PPP1R21-DT, PRC1, PRCD, PRELP, PRKAR2B, PRR11, PRR14, PRR16, PRRT1, PRSS12, PRSS23, PRSS35, PSMB8, PTCH1, PTGDR2, PTGER2, PTGS1, PTK7, PTN, PTPN18, PXK, PXMP4, PYROXD2, RAB11B-AS1, RAB11FIP3, RAB31, RAB33B-AS1, RAB37, RAB40B, RACGAP1, RARG, RARRES2, RASL10B, RASSF8-AS1, RBMS3-AS3, RCAN2, RCBTB2, RDH5, RELA-DT, RENO1, RERG

RFC3, RFC5, RFTN1, RHBDL1, RHOBTB2, RHOBTB3, RHOJ, RHOXF1-AS1, RIN2, RIPOR3, RIPOR3-AS1, RMI2, RORB, RPLP1P6, RPS6KA2, RTL8B, RUNDC3A-AS1, RUNX1T1, RUSC1, RXFP1, S100A3, S100A4, S1PR3, SALL2, SAMD5, SAMHD1, SAMMSON, SARM1, SBF2-AS1, SCN1B, SCNN1A, SCUBE1, SDC3, SEC31B, SELENBP1, SELENON, SELENOP, SEMA6A, SENP8, SEPTIN4, SEPTIN8, SEPTIN9, SESN1, SFRP1, SGO2, SH3BGRL, SHC4, SHCBP1, SHISA3, SHROOM3, SIDT2, SIPA1, SIRT3, SIX2, SKA1, SLC16A12, SLC16A4, SLC19A1, SLC1A7, SLC20A1-DT, SLC25A12, SLC25A23, SLC2A12, SLC35C1, SLC38A4, SLC40A1, SLC44A1, SLC44A3, SLC4A4, SLC66A3, SLC7A7, SLC9A3-AS1, SLITRK5, SLITRK6, SMAD3, SMAD6, SMAD9, SMARCD2, SMC2, SMC4, SMIM10, SMPDL3A, SNAP25, SNED1, SNHG31, SNTB2, SOCAR, SOCS2, SOX12, SOX13, SP2-DT, SPAAR, SPATA33, SPATA6, SPC24, SPC25, SPIRE2, SPRY1, SPTSSA, SRPX, SRPX2, SSBP2, SSBP3, SSBP3P1, SSR4P1, ST20-AS1, ST3GAL5, ST8SIA1, STAT6, STING1, STK32B, STMN1, STN1, STOM, STON1, STS, STX18-AS1, SUFU, SUGCT, SULF2, SVBP, SYNC, SYNPO, SYNPO2, SYP, SYT1, SYTL2, TACC3, TAGLN2, TARID, TBXAS1, TCAF2, TCF19

TCF21, TCF4, TEAD2, TEF, TEK, TELO2, TGFBR2, THRA, TIMP2, TK2, TLCD2, TLE2, TMCO6, TMEM107, TMEM109, TMEM119, TMEM129, TMEM14A, TMEM150C, TMEM19, TMEM240, TMEM37, TMEM44, TMEM51, TMEM51-AS1, TMEM52B, TMEM64, TMEM80, TMEM8B, TMEM98, TMOD2, TMSB15A, TMTC4, TMX4, TNFAIP8, TNFRSF11B, TNFRSF19, TNFRSF1A, TNFRSF1B, TNFRSF21, TNS1, TNS2, TNS3, TOB2, TOGARAM2, TOP2A, TOR3A, TOX, TPCN2, TPRG1-AS1, TPTE2P1, TPX2, TRAPPC9, TRHDE-AS1, TRIM65, TRIM8, TRIOBP, TRIP13, TRPA1, TRPC3, TSC22D1-AS1, TSC22D3, TSPAN11, TSPAN12, TSPAN15, TSPAN9, TSPY26P, TSTD3, TTC28, TTK, TTLL3, TWIST1, TWSG1, TWSG1-DT, TXNIP, TXNRD2, TXNRD3, TYMS, TYMSOS, UACA, UBE2C, UBL7-DT, UBTF, ULK2, UNC5C, UNC93B1, UQCC3, VAC14-AS1, VAMP5, VASH1, VAT1L, VCAN, VNN2, VPS9D1-AS1, VWA5A, WARS2-IT1, WDR90, WNT2B, XRCC1, XXYLT1, YPEL3, ZBTB16, ZBTB47, ZBTB47-AS1, ZC3H7B, ZCCHC24, ZFP36L2, ZFYVE21, ZHX3, ZMAT3, ZMYM3, ZNF185, ZNF337-AS1, ZNF362, ZNF385A, ZNF395, ZNF467, ZNF503, ZNF536, ZNF608, ZNF703, ZNF710-AS1, ZNF883, ZSCAN16-AS1, ZWILCH, ZWINT

Upregulated

AAK1, AARS1, ABHD17B, ABHD3, ABHD4, ABL2, ABTB2, ACTR6, ADAMTS6, ADNP2, ADPRHL1, AFG3L1P, AFTPH, AGTPBP1, AGTR1, AHI1, AHSA1, AIFM2, AKAP17A, AKR1C2, AKR1C7P, ALAS1, ALKBH3, AMIGO2, AMMECR1L, ANKRD1, ANKRD42, ANXA1, AP4B1, AP5Z1, APOOL, ARAP2, ARG2, ARHGAP26, ARHGEF35-AS1, ARL4C, ARL8A, ARNTL2, ASAH2B, ASB1, ASIC3, ATF3, ATG4A, ATMIN, ATP13A3, ATP1B1, ATP2B1, ATP6V0A1, ATP6V0A2, ATP6V0B, ATP6V0D2, ATP6V1B2, ATP6V1C1, ATP6V1G1, ATRNL1, B4GALNT1, BACH1-IT1, BAG3, BANP, BCAS2, BCL10, BCL7A, BEND7, BEX2, BEX4, BHLHA15, BHMT2, BICD1, BIRC3, BLZF1, BMP2, BRF2, BRICD5, BRPF1, BSDC1, BTG3, BTN2A1, BTN2A2, C10orf88, C17orf107, C17orf67, C18orf25, C19orf47, C1orf52, C2CD2L, C3orf52, C6orf141, C6orf62, C8orf88, C9orf72, CA13, CACYBP, CACYBPP2, CAMSAP1, CAP2, CASP3, CASP4LP, CCDC174, CCDC30, CCDC47, CCDC59, CCDC82, CCDC88C, CCDC9, CCL20, CCL26, CCN1, CCN2, CCNE1, CCNK, CCNL1, CCT2, CCT4, CCT6A, CCZ1P1, CD163L1, CD274, CD40, CD55, CD58, CD99P1, CDCP1, CDH3, CECR2, CENATAC, CEP170B, CEP290, CFAP20, CFAP251, CFAP53

CGAS, CHD1, CHD7, CHORDC1, CHRNA5, CHST6, CIRBP-AS1, CKMT2, CLCF1, CLDN1, CLDN4, CLEC2D, CLIC2, CLINT1, CLU, CMKLR2, CNKSR3, CNNM4, CNST, COG3, COP1-DT, COQ10B, COX19, CPEB1, CPEB4, CPNE2, CPNE8, CREB5, CRELD2, CREM, CRY1, CRYAB, CRYM-AS1, CSNK2A1, CSRNP1, CSRP2, CTAGE4, CTH, CTPS1, CWC25, CXADR, CXCL2, CXCL3, CXCL5, CXCL8, CYB5R1, CYB5R4, CYLD, CYLD-AS1, DAP3, DCAF1, DCDC2C, DCLK2, DCP1A, DCUN1D5, DDIT3, DDX21, DDX27, DDX55, DEDD2, DERL3, DGKG, DHDDS, DHX34, DLC1, DNAH14, DNAH17, DNAJA1, DNAJA4, DNAJB1, DNAJB11, DNAJB4, DNAJB9, DNAJC2, DNAJC24, DNAJC3, DNAJC6, DNAL1, DNHD1, DNM1, DNTTIP2, DOCK10, DOLPP1, DOT1L, DPH3, DUS4L, DUSP1, DUSP12, DUSP5, DYNC1I1, DYRK3, E2F3, E2F5, E2F6, EAF1, EAF2, EDN1, EFCAB7, EFL1, EFNB2, EIF1AD, EIF1AY, EIF1B, EIF2AK3, EIF5, ELOVL7, ENTPD7, EPB41, EPDR1, EPHA2, EPHA5, EPS15L1, ERCC8, ERN1, ERO1B, ETV5, EVC2, F2RL2, FAF2, FAM126B, FAM174A, FAM177B, FAM210A, FAM217B, FAM219A, FAM83G, FBXO34

FGF2, FGF5, FGFR1OP2, FICD, FKBP4, FLNB, FLNC, FLNC-AS1, FNBP4, FNDC5, FXR1, GAB2, GABPB1, GABRE, GADD45A, GAN, GARS1, GAS8, GBE1, GCC1, GCFC2, GCLM, GCNT3, GDAP2, GDF15, GDPD1, GEM, GEMIN7-AS1, GFM2, GFPT1, GHITM, GID4, GIPR, GJC1, GK, GKAP1, GLA, GLRB, GLRX3, GOLGA6L9, GOLPH3L, GOLT1B, GORAB, GOSR2, GOT1, GPER1, GPN1, GPRASP1, GRPEL2, GSDMB, GTF2B, GTF2IP20, GTF3C3, GTPBP2, GTPBP4, GUCA1B, GUCY1A2, GUSBP1, H19, H2BC15, HAGLR, HBEGF, HCCS, HCG18, HDAC9, HECW2, HELB, HERC2P9, HERPUD1, HES1, HEY1, HGS, HIF1A-AS3, HINFP, HK2, HKDC1, HMBOX1, HMGA2, HMOX1, HOXD1, HSD17B7, HSF2, HSP90AA1, HSP90AA2P, HSP90AA3P, HSP90AB1, HSP90AB3P, HSP90B1, HSPA12A, HSPA13, HSPA1A, HSPA1B, HSPA4, HSPA4L, HSPA5, HSPA5P1, HSPA7, HSPA8, HSPA8P1, HSPA9, HSPB1P1, HSPB8, HSPBAP1, HSPD1, HSPD1P1, HSPE1, HSPH1, HYOU1, ICAM1, IER3, IFRD1, IGF2R, IGHMBP2, IL11, IL12A, IL32, IL4I1, IL6, IL7R, INHBA, INHBB, INO80C, INTU, IPPK, IQCB1, IRAK2, IRGQ, ITPRIP, JKAMP, JMJD1C, JMJD6, KBTBD8, KCMF1, KCNE4, KCNK1, KCNQ5, KCTD5

KIAA0319, KIF1A, KIF21A, KIF9-AS1, KIZ, KLF2, KLF5, KLF6, KRT34, LAMA1, LARP1B, LARP6, LDAF1, LGALS8, LHFPL2, LHFPL3-AS2, LIF, LIMS1, LIN52, LINC00520, LINC00632, LINC00680, LINC00888, LINC01121, LINC01705, LINC02154, LINC02392, LINC02605, LINC02693, LINC-PINT, LMTK2, LONP1, LRIF1, LRP8, LRRC15, LRRC4, LRRC47, LRRC49, LRRFIP1, LRRFIP2, LSG1, LTO1, LTV1, LURAP1L, M6PR, MACO1, MAFF, MAFG, MAGOH, MAK16, MANF, MAP1B, MAP1LC3B, MAP1LC3B2, MAP2, MAP3K9, MAPK8, MAPRE2, MARCHF3, MATN1-AS1, MDM4, ME2, MED10, MED26, MED31, MED8, MET, METTL4, MFSD2A, MFSD9, MIA2, MIA3, MICA, MICB, MIR100HG, MIR137HG, MIR22HG, MIR31HG, MIRLET7A1HG, MIS12, MLLT11, MMADHC-DT, MMP1, MMP10, MOAP1, MOCOS, MOK, MOSMO, MPHOSPH10, MRAS, MREG, MRPL18, MSC, MSTO1, MSX1, MT1A, MT1E, MT1F, MT1G, MT1M, MT1X, MTF1, MTFR1, MTHFD2L, MTRF1, MT-RNR2, MXD1, MYCT1, MYEF2, NAA25, NAA60, NAF1, NAIF1, NAP1L2, NAV2-AS6, NAV3, NBPF25P, NBPF8, NCF2, NDEL1, NEAT1, NECAP1, NEDD4L, NETO1, NFKB2, NFKBIZ, NGDN, NGLY1, NHLH1, NIBAN1, NKX3-1, NMRAL2P, NOCT, NPAS2, NPC1, NPIPB2, NPIPB4

NQO2, NRIP3, NSFL1C, NSRP1, NSUN3, NUCB2, NUDC, NUS1, NUS1P1, NXF1, NXPE3, OCLN, OR2A9P, ORC3, OSBPL6, OSER1, OSGIN2, OTUD1, OTUD7A, OTULIN, OXNAD1, PACC1, PALM3, PAPOLA, PAPPA2, PBX4, PCDH10, PDIA3, PDIA4, PDLIM5, PGLS-DT, PGM3, PHACTR1, PHEX, PHKG1, PHLDA1, PIGA, PIK3CD, PIM3, PITHD1, PKD1P6, PLA2G4C, PLAA, PLCXD1, PLD6, PLEK2, PLEKHA3, PLEKHB2, PLIN2, PLK3, PLRG1, PMAIP1, PMS2CL, PNLIPRP3, PNP, POC5, PODXL, POLR3A, POLR3E, POLR3F, POLR3G, POM121, POM121C, POU5F1, PPID, PPIF, PPP1R15A, PPP1R26, PPP2R2C, PPP3CC, PRDM4, PRELID3A, PRELID3B, PRH1, PRMT5, PSMA3, PSMA4, PSMA6, PSMA6P1, PSMB2, PSMC1, PSMC4, PSMC6, PSMD11, PSMD12, PSMD13, PSMD14, PSMD2, PSMD3, PSMD6, PSME4, PTAFR, PTER, PTGES3, PTGS2, PTOV1-AS2, PTPRB, PTX3, PVR, PYROXD1, QRSL1, RAB33A, RAB3IP, RABGGTB, RAD54L2, RAD9A, RAE1, RALA, RANBP10, RAPGEFL1, RASAL2, RBBP6, RBM15, RBM19, RBM24, RCAN1, RCL1, RELB, RELT, REPS1, RGS7, RHBDD3, RHEBL1, RINT1, RIPK2, RIPK2-DT, RIT1, RND3, RNF144B, RNF149, RNF4, RNF6, RNFT1, RNU6-9, ROR1-AS1, RP9P

RPLP0P2, RPS6KA1, RPS6KC1, RRAD, RRAGD, RRP12, RTCA, RUBCN, RUSC2, SAMD4B, SAT1, SBDSP1, SCAND2P, SCARNA7, SCML1, SCN1A-AS1, SDAD1, SDE2, SDF2L1, SEC11C, SEC14L1P1, SEC22B4P, SEC23B, SEC61A2, SELENOI, SELENOK, SELENOS, SENP5, SEPHS2, SEPTIN7P14, SEPTIN7P2, SERP1, SERPINB8, SESN2, SETD4, SFMBT1, SGCG, SGTB, SH3BP5, SH3BP5L, SH3RF1, SLC10A7, SLC19A2, SLC20A1, SLC22A1, SLC22A15, SLC22A5, SLC25A25, SLC25A33, SLC26A11, SLC2A3, SLC30A1, SLC30A6, SLC31A1, SLC33A1, SLC39A14, SLC3A2, SLC43A3, SLC45A3, SLC45A4, SLC4A1AP, SLC7A1, SLC7A5, SMIM13, SMN1, SMN2, SMO, SNAPC1, SNIP1, SNX10, SOWAHC, SP140, SPATA2, SPATA4, SPATS2, SPCS2P4, SPHK1, SPIRE1, SPRED3, SPRY4, SPX, SQSTM1, SRP19, SRP54, SS18L1, SSR3, ST3GAL6, STAM2, STARD8, STIP1, STOX2, STRIP2, STX16, STX3, STYK1, SUCO, SUDS3, SUGCT-AS1, SUGT1, SULT1C2, SUPT6H, SUPV3L1, SYVN1, TACC2, TAF13, TAF1A, TAGLN3, TANGO2, TATDN1, TBP, TBX3, TCP1, TDG, TDP2, TDRKH, TDRP, TENT4B, TFG, TGFB2, TGIF1, THAP5, THUMPD2, TIRAP, TM6SF1, TMEM154, TMEM199, TMEM33, TMEM38B, TMEM39A, TMEM41B, TNFAIP3, TNFAIP6, TNFRSF10A, TNFRSF10B, TNFSF9, TOP3A, TP53BP2

TPMT, TRAF2, TRAF3, TRIB1, TRIB3, TRIM26, TRMT10A, TSLP, TSPYL2, TTBK2, TTC1, TTC9, TTLL7, TUBA4A, TUBB4A, TUBE1, TUBG1P, TUFT1, TVP23B, TVP23C, TXNDC11, TXNL1, TXNL4B, TYW3, UAP1, UBASH3B, UBB, UBBP4, UBC, UBE2O, UBQLN1, UBXN4, UFD1, UFM1, UGCG, ULBP1, ULBP2, ULBP3, ULK4, UNKL, UPP1, USP14, USP36, USP38, USP53, UTP11, VCPKMT, VEGFC, VEZT, VLDLR, VMP1, VPS37A, WBP2, WHRN, XBP1, XIAP, YKT6, YOD1, YRDC, YWHAG, ZBTB49, ZCCHC4, ZCCHC7, ZCCHC8, ZDHHC18, ZFAND2A, ZFAND5, ZFPM2-AS1, ZNF143, ZNF195, ZNF202, ZNF222, ZNF26, ZNF267, ZNF274, ZNF275, ZNF316, ZNF365, ZNF419, ZNF433, ZNF473, ZNF547, ZNF554, ZNF558, ZNF560, ZNF562, ZNF613, ZNF674-AS1, ZNF697, ZNF761, ZNF773, ZNF778, ZNF804A, ZNF823, ZPR1, ZRSR2, ZSCAN20, ZSCAN5A, ZUP1

Whole transcriptome RNA sequencing of CD-HIFs 24 hours

Supplementary Table 8

Ileal histology scores, fibrosis scores, and ileal gene expression (fold)

	HS score	FS score	Col1a2 mRNA	Col3a1 mRNA	Zeb1 mRNA	Vim mRNA	Acta2 mRNA	Tnf mRNA	Emr1 mRNA
42-week-old AKR untreated	0.00	0.00	1.12	1.11	1.10	1.10	1.30	1.07	1.10
42-week-old SAMP1Yit/Fc + H2O	11.80	2.80	20.33	23.57	21.90	482.75	2.31	2.05	4.42
42-week-old SAMP1Yit/Fc + Fidaxomicin	2.83	0.67	20.29	5.57	11.10	157.54	0.31	0.24	0.69
42-week-old SAMP1Yit/Fc + anti-PDGFRβ antibody	3.20	0.96	0.49	5.91	0.27	0.29	0.38	0.18	0.22
42-week-old SAMP1Yit/Fc + Fidaxomicin + Pdgfrb-OE-LV	9.25	2.50	18.94	13.52	10.28	77.99	3.98	3.15	7.96
42-week-old SAMP1Yit/Fc + Gsk3b-siRNA-LV	3.51	0.83	0.58	0.12	0.27	0.14	0.08	1.66	0.06
42-week-old SAMP1Yit/Fc + Fidaxomicin + Gsk3b-OE-LV	9.18	2.60	19.88	12.30	11.30	90.20	4.40	1.54	5.50

Ileal overall disease activity calculation (%)

	HS score	FS score	Col1a2 mRNA	Col3a1 mRNA	Zeb1 mRNA	Vim mRNA	Acta2 mRNA	Tnf mRNA	Emr1 mRNA	% ODA
42-week-old AKR untreated	0	0	0	0	0	0	0	0	0	0
42-week-old SAMP1Yit/Fc + H2O	100	100	100	100	100	100	100	100	100	100
42-week-old SAMP1Yit/Fc + Fidaxomicin	24	24	100	24	51	33	13	12	16	33
42-week-old SAMP1Yit/Fc + anti-PDGFRβ antibody	27	34	2	25	1	0	16	9	5	13
42-week-old SAMP1Yit/Fc + Fidaxomicin + Pdgfrb-OE-LV	78	89	93	57	47	16	172	154	180	99
42-week-old SAMP1Yit/Fc + Gsk3b-siRNA-LV	30	30	3	0	1	0	3	81	1	17
42-week-old SAMP1Yit/Fc + Fidaxomicin + Gsk3b-OE-LV	78	93	98	52	52	19	190	75	124	87

Supplementary Table 9

Changes in body weight from week 40 to week 42	
SAMP1/YitFc	99%
SAMP1/YitFc + Fidaxomicin	94%
SAMP1/YitFc + anti-PDGFRβ antibody	91%
SAMP1/YitFc + Fidaxomicin Pdgfrb-OE-LV	97%
SAMP1/YitFc + Gsk3b-siRNA-LV	93%
SAMP1/YitFc + Fidaxomicin + Gsk3b-OE-LV	102%

Supplementary Table 10

Target Name	ChEMBL-ID	UniProt ID	PDB Visualization	TTD ID	Probability	Model accuracy	
DNA-(apurinic or apyrimidinic site) lyase	CHEMBL5619	P27695	6BOW	T13348	99.71%	91.11%	
Cannabinoid CB2 receptor	CHEMBL253	P34972	6KPF	Not Available	98.32%	97.25%	
Dual specificity protein kinase CLK4	CHEMBL4203	Q9HAZ1	6FYV	Not Available	97.92%	94.45%	
Nuclear factor NF-kappa-B p105 subunit	CHEMBL3251	P19838	1SVC	Not Available	97.16%	96.09%	NF-kB
Kruppel-like factor 5	CHEMBL1293249	Q13887	Not Available	Not Available	96.13%	86.33%	
Cathepsin D	CHEMBL2581	P07339	4OD9	T67102	96.12%	98.95%	
Cannabinoid CB1 receptor	CHEMBL218	P21554	6N4B	Not Available	95.12%	96.61%	
Pregnane X receptor	CHEMBL3401	O75469	6TFI	T82702	94.83%	94.73%	
PI3-kinase p110-delta subunit	CHEMBL3130	O00329	6PYR	T67849	92.8%	96.47%	
Platelet-derived growth factor receptor alpha	CHEMBL2007	P16234	7LBF	T53524	91.98%	91.07%	PDGFRA
LSD1/CoREST complex	CHEMBL3137262	O60341	5L3D	Not Available	90.49%	97.09%	
Heat shock protein HSP 90-beta	CHEMBL4303	P08238	5FWK	Not Available	90%	96.77%	
Voltage-gated N-type calcium channel alpha-1B subunit	CHEMBL4478	Q00975	Not Available	T38338	89.6%	97.14%	
PI3-kinase p110-beta subunit	CHEMBL3145	P42338	Not Available	T05031	88.07%	98.75%	
Hypoxia-inducible factor 1 alpha	CHEMBL4261	Q16665	4H6J	Not Available	86.81%	85.14%	
NT-3 growth factor receptor	CHEMBL5608	Q16288	6KZD	Not Available	86.35%	95.89%	
Transcription intermediary factor 1-alpha	CHEMBL3108638	O15164	4YBM	Not Available	86.33%	95.56%	
Cyclooxygenase-1	CHEMBL221	P23219	6Y3C	Not Available	85.57%	90.17%	
Dual specificity protein phosphatase 3	CHEMBL2635	P51452	3F81	Not Available	85.06%	94%	
G-protein coupled receptor 6	CHEMBL3714130	P46095	Not Available	Not Available	85%	97.36%	
Proteasome component C5	CHEMBL4208	P20618	6KWY	Not Available	85%	90%	
Toll-like receptor 4	CHEMBL5255	O00206	4G8A	T81443	84.64%	92.5%	
Casein kinase II alpha/beta	CHEMBL3038477	P67870	6TLS	T51565	84.35%	99.23%	
IgG receptor FcRn large subunit p51	CHEMBL5966	P55899	6FGB	Not Available	84.24%	90.93%	
Indoleamine 2,3-dioxygenase	CHEMBL4685	P14902	6E43	T89697	82.99%	96.38%	
Protein-tyrosine phosphatase 1B	CHEMBL335	P18031	5QGF	Not Available	82.92%	95.17%	
Thyroid hormone receptor alpha	CHEMBL1860	P10827	3ILZ	T79591	82.23%	99.15%	
Adenosine A1 receptor	CHEMBL226	P30542	5N2S	T92072	82.11%	95.93%	
C5a anaphylatoxin chemotactic receptor	CHEMBL2373	P21730	6C1R	T15439	82.09%	92.62%	
DNA topoisomerase II alpha	CHEMBL1806	P11388	6ZY5	T17048	81.89%	89%	
Protein-tyrosine phosphatase 2C	CHEMBL3864	Q06124	5EHR	T13057	79.7%	94.42%	
Lysosomal Pro-X carboxypeptidase	CHEMBL2335	P42785	3N2Z	Not Available	79.52%	100%	
Plasminogen activator inhibitor-1	CHEMBL3475	P05121	3CVM	T15556	79.09%	83%	
Cytochrome P450 3A4	CHEMBL340	P08684	5VCC	T37848	78.94%	91.19%	
Cyclooxygenase-2	CHEMBL230	P35354	5F19	Not Available	78.68%	89.63%	
Nuclear receptor ROR-beta	CHEMBL3091268	Q92753	Not Available	Not Available	78.67%	95.5%	
Tissue factor pathway inhibitor	CHEMBL3713062	P10646	5NMV	T78890	78.63%	97.33%	
Platelet-derived growth factor receptor	CHEMBL2095189	P09619	3MJG	T53524	77.99%	71.67%	PDGFR

Supplementary Table 11

Percentage of serum proteins, relative to healthy donors

Serum Proteins	Healthy	CDS	CDNS
PDGF R alpha	100	98	141
PDGF R beta	100	125	167
PDGF-AA	100	155	77
PDGF-AB	100	134	105
PDGF-BB	100	116	95
PDGF-C	100	92	81
PDGF-D	100	112	87
SPARC	100	81	67

Supplementary Figure 1

Molecular docking of Fidaxomicin and PDGFR by CB-Dock2

A

C1 Vina score -7.6

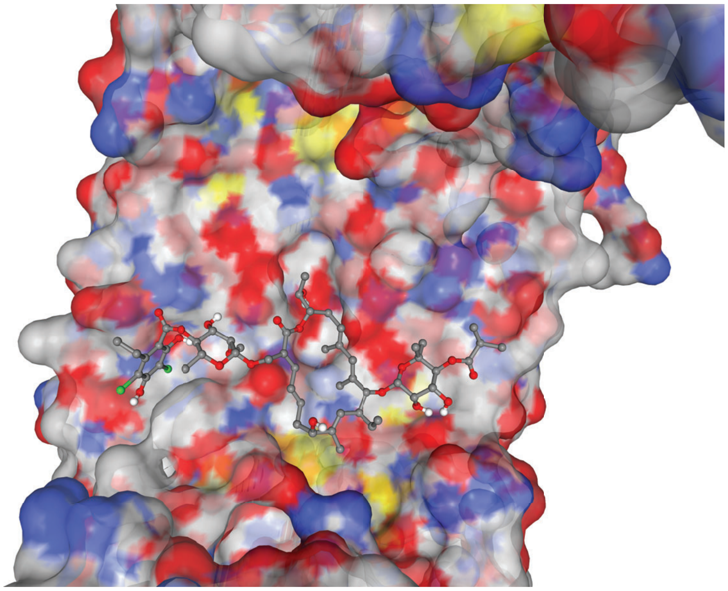

C2 Vina score -6.7

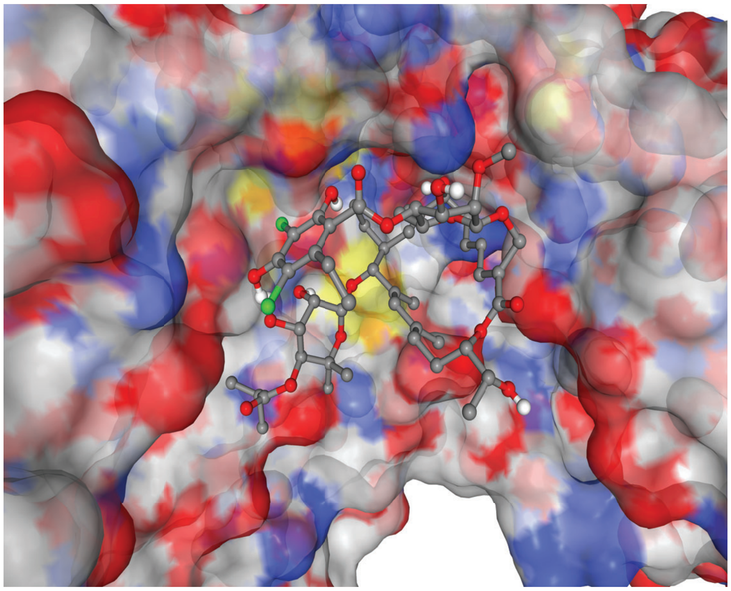

C3 Vina score -8.1

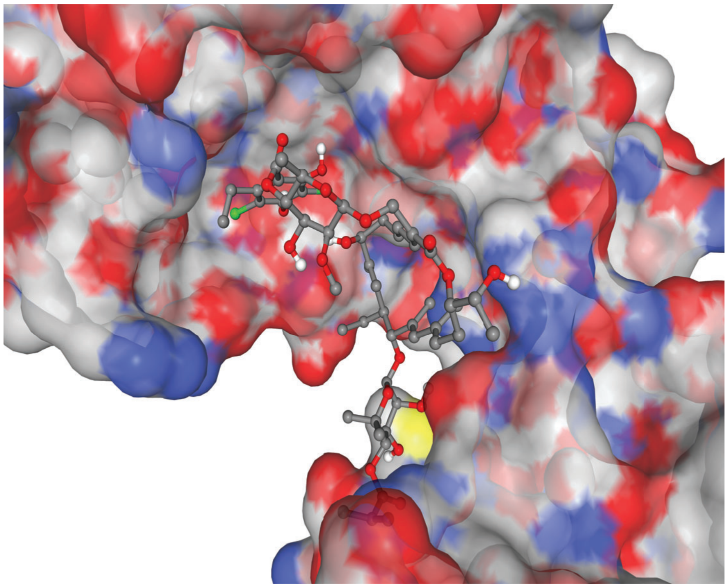

C4 Vina score -8.5

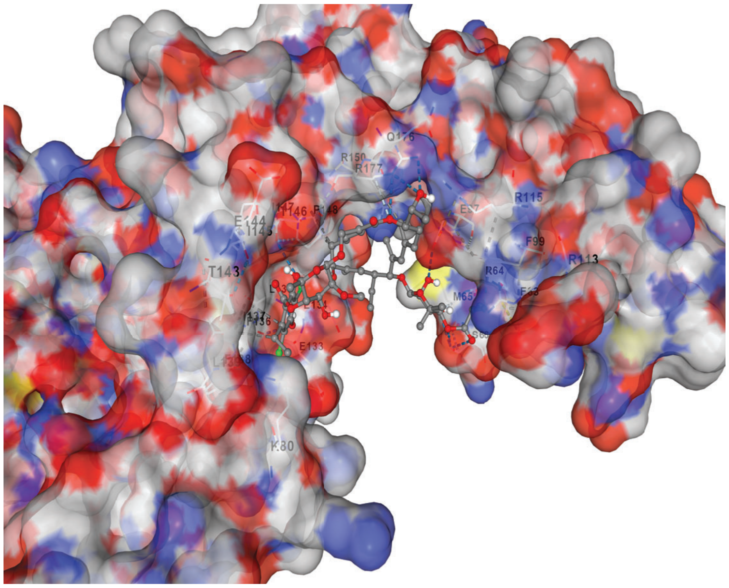

C5 Vina score -7.4

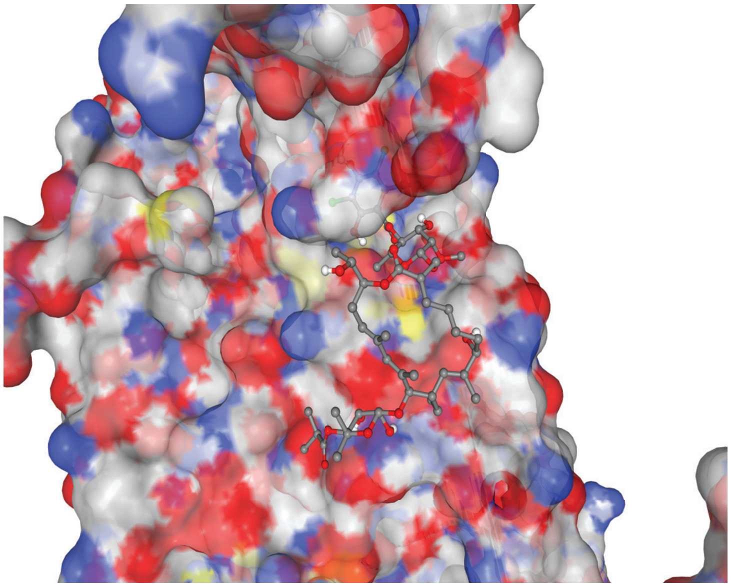

B

CB-Dock2 analysis (Auto BlindDock)

Best Vina score = -8.5 kcal/mol

CurPocket ID	Vina score	Cavity volume (Å^3)	Center (x, y, z)	Docking size (x, y, z)
⦿ C4	-8.5	521	22, 4, -21	33, 33, 33
○ C3	-8.1	568	16, -53, 35	33, 33, 33
○ C1	-7.6	1082	18, -49, 5	33, 33, 33
○ C5	-7.4	432	11, -39, 12	33, 33, 33
⦿ C2	-6.7	609	22, -29, -18	33, 33, 33

Supplementary Figure 2

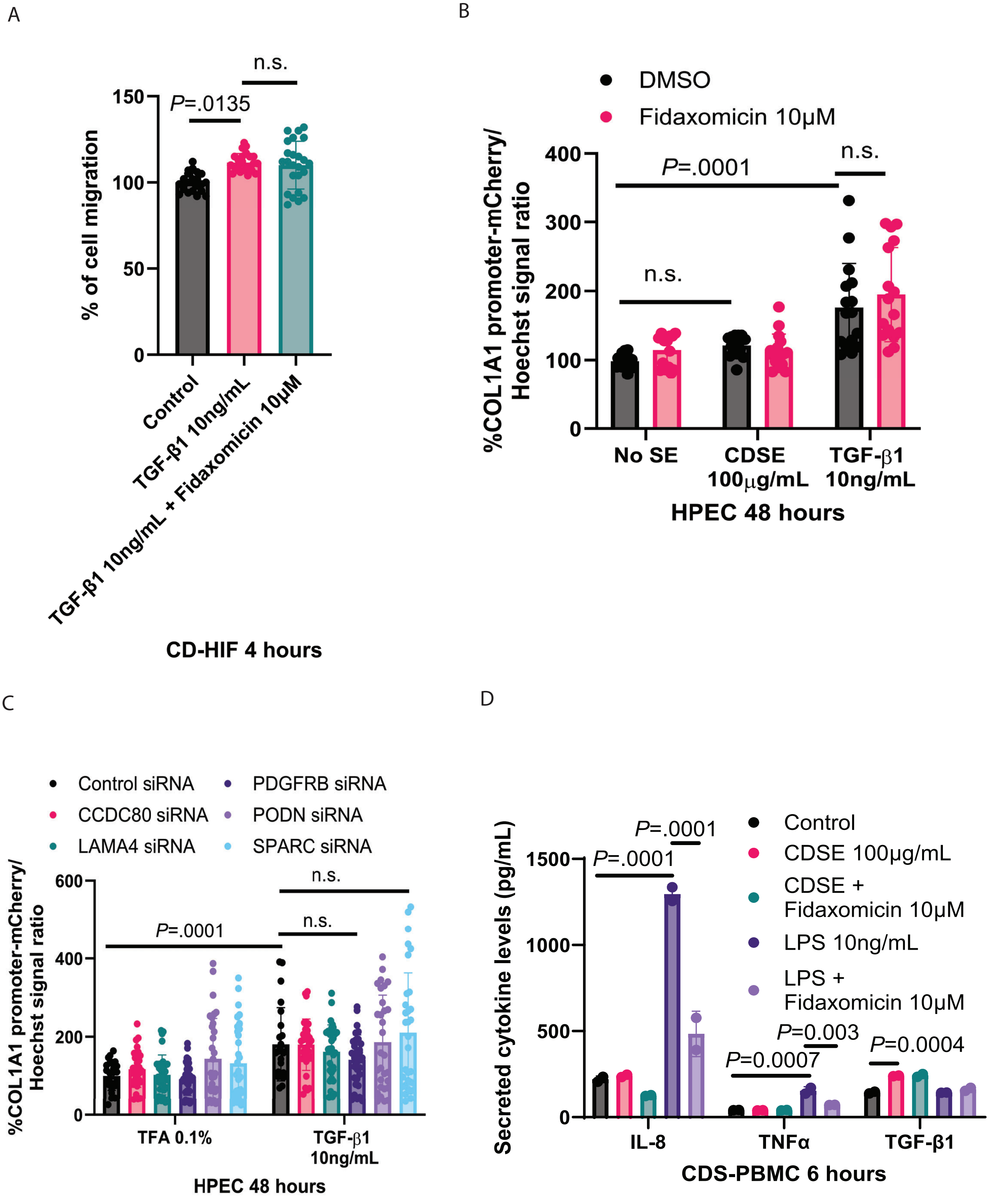

Supplementary Figure 3

A

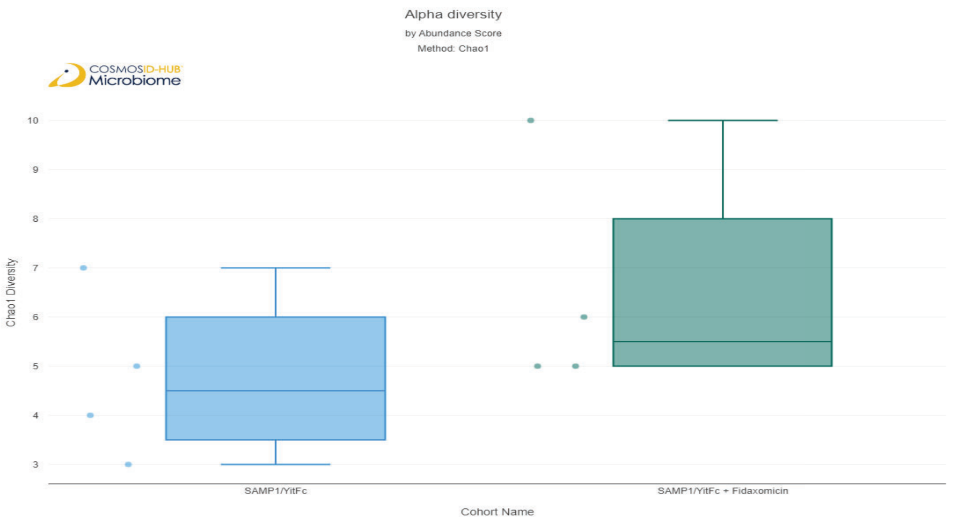

B

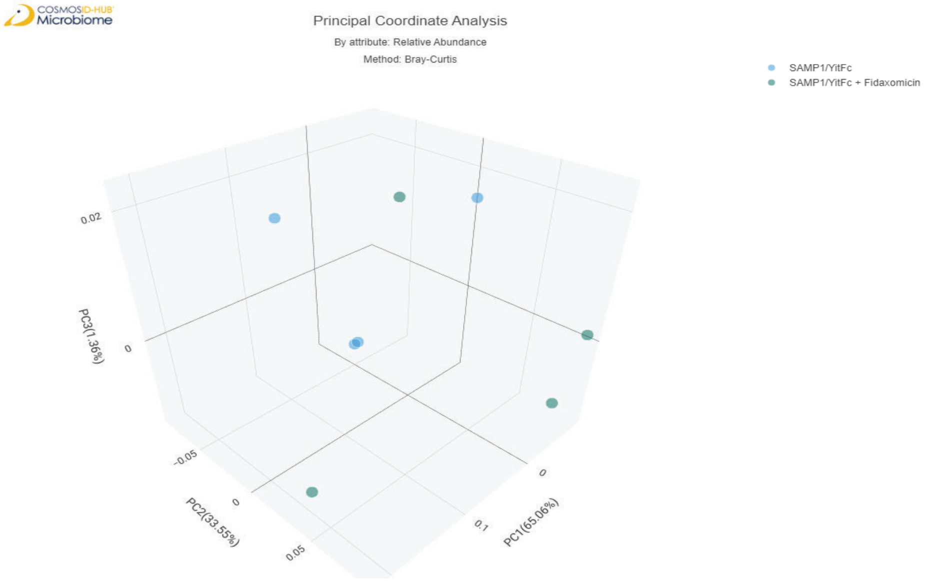

C

Mouse ileal relative abundance top 12 bacterial species

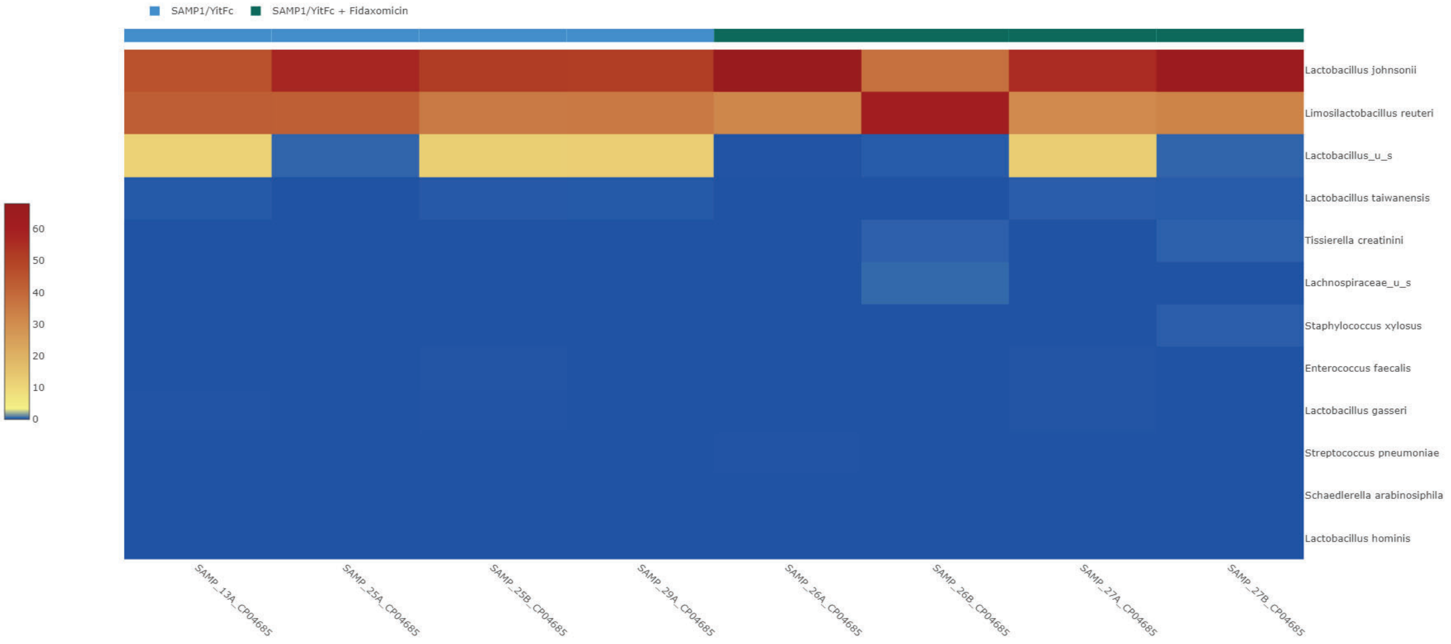

Supplementary Figure 4

A

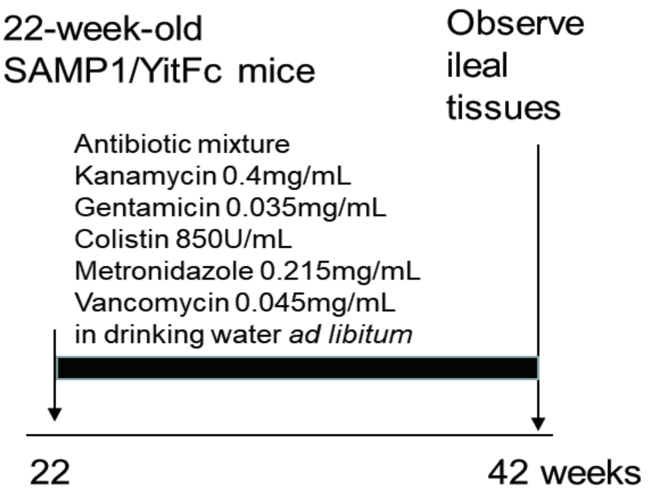

B

SAMP1/YitFc + ABX

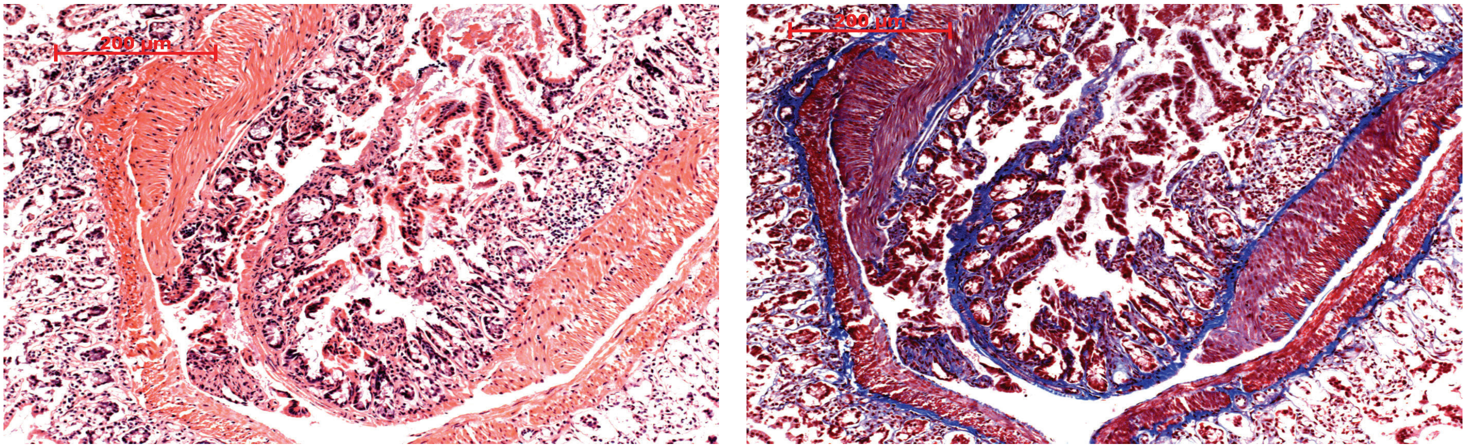

HE Mouse ileal tissues 100X magnification MT

C

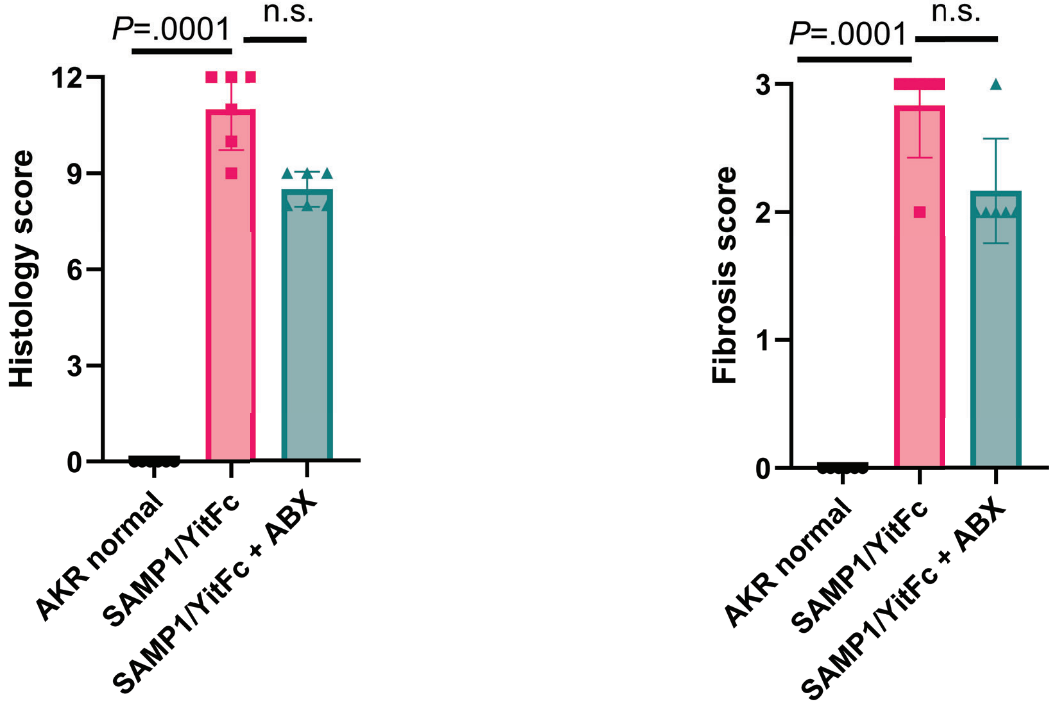

Supplementary Table and Figure Legends:

Supplementary Table 1

Baseline characteristics

Baseline characteristics of 7 CDS blood sample donors.

Supplementary Table 2

Baseline characteristics

Medical notes of 4 fresh CDS ileal explant donors and a fresh non-IBD ileal explant donor from UCLA Pathology.

Supplementary Table 3

Baseline characteristics

Baseline characteristics of 4 donors of CD-HIFs and HPECs.

Supplementary Table 4

10X Genomics Visium spatial RNA sequencing data.

The significantly up-regulated genes in 5 fibrotic clusters compared to non-fibrotic clusters are shown.

Each fibrotic cluster was identified by significantly higher collagen mRNA expression than adjacent non-fibrotic clusters with $P<.0001$.

CD02 clusters 4 and 8 had high COL1A1, COL1A2, and COL3A1 mRNA expression.

CD04 cluster 2 had high COL12A1 mRNA expression.

CD05A cluster 1 had high COL12A1, COL15A1, and COL3A1 mRNA expression.

CD05A cluster 2 had high COL3A1 mRNA expression.

n=3 CDS patients (CD02, CD04, and CD05A). Two patients (CD02 and CD05A) had two fibrotic clusters.

The full dataset can be found here: DOI: 10.5061/dryad.ncjsxkt6q

Supplementary Table 5

Hit list of HTS.

HTS of 2621 FDA-approved drugs with CDSE-treated CD-HIFs. The drugs that inhibited COL1A1 promoter activity by 2 SDs are shown.

Supplementary Table 6

Fidaxomicin may be a target of PDGFR.

Super-PRED target prediction analysis of drugs in the FDA-approved hit list. The predicted probability of binding to PDGFR, PDGFRA, and PDGFRB and their known targets are shown. Midostaurin and Ponatinib are known PDGFR binders. Fidaxomicin has a very high probability of being a PDGFR target. Target information was provided by the datasheet of the FDA-approved drug library.

The Super-PRED website is https://prediction.charite.de/

Supplementary Table 7

Fidaxomicin inhibited PDGFRB and collagen mRNA expression in intestinal fibroblasts.

Whole-transcriptome RNA sequencing. CD-HIFs were treated with 100μg/mL CDSE for 30 minutes, followed by 0.8% DMSO or 10μM fidaxomicin for 24 hours. The left side shows the difference in gene expression with and without CDSE. The right side shows the difference in gene expression in CDSE-treated CD-HIFs with and without fidaxomicin. The orange color highlighted significantly increased genes. The green color highlighted significantly decreased genes. Results were pooled from four experiments. PDGFRB (highlighted in red) mRNA expression was reduced in CDSE-treated CD-HIFs.

The full dataset can be found here: DOI: 10.5061/dryad.ncjsxkt6q

Supplementary Table 8

Fidaxomicin reduced mRNA expression of ileal fibrogenic and inflammatory genes in SAMP1/YitFc mice.

For real-time RT-PCR data, mouse ileal mRNA expression was normalized to endogenous control Gapdh. Average values of fold-change are displayed. Ileal histology score (HS), ileal fibrosis score (FS), and ileal mRNA expression were converted into percentages. Average values are shown. ODA values are the average values of all parameters. Fidaxomicin, anti-PDGFRβ antibody, and Gsk3b-siRNA lentivirus reduced ileal ODAs. Lentiviral Pdgfrb and Gsk3b overexpression abolished the inhibitory effect of fidaxomicin. N=6 mice per group.

Col1a2 = Collagen 1A2

Col3a1 = Collagen 3A1

Zeb1 = Zinc Finger E-box Binding Homeobox 1

Vim = Vimentin

Acta2 = smooth muscle alpha-2 actin

Tnf = Tumor necrosis factor

Emr1 = EGF-like module-containing mucin-like hormone receptor-like 1 or F4/80 or Adgre1

Supplementary Table 9

Oral fidaxomicin treatment mildly reduced body weight in SAMP1/YitFc mice.

Changes in body weight from 40 to 42 weeks of age of SAMP1/YitFc mice. Fidaxomicin, anti-PDGFRβ neutralizing antibody, and Gsk3b-siRNA-lentivirus treatment caused mild weight loss in SAMP1/YitFc mice. The fidaxomicin-mediated weight loss was abolished by lentiviral Pdgfrb and Gsk3b overexpression. Average values of percentages are shown.

Supplementary Table 10

Predicted binding targets of fidaxomicin.

Super-PRED prediction of potential binding targets of fidaxomicin. The predicted binding targets are shown, including NF-κB, PDGFA, and PDGF.

Supplementary Table 11

Serum levels of PDGFR-related proteins and SPARC are not affected by CD strictures.

Serum proteomics of healthy donors, CDS patients, and CDNS patients. The serum samples from 4 donors per group were pooled and sent to RayBiotech Company for full human L-type array testing service. The arrays detected 1000 proteins. After normalization with control signals, the levels of serum proteins were converted to percentages relative to those from healthy donors. Average values are shown. No significant differences in the listed proteins were found among CDS and CDNS patients compared to healthy donors.

The full dataset can be found here: DOI: 10.5061/dryad.ncjsxkt6q

Supplementary Figure 1

Molecular docking prediction analysis of fidaxomicin and PDGFR by CB-Dock2.

The PDGFR (3MJG) and fidaxomicin (FI8) structures were found in the RCSB PDB database.

(**A**) The images of 5 predicted PDGFR binding sites for fidaxomicin with negative Vina scores.

(**B**) A table of analysis results. Negative vina scores (kcal/mol) indicated the high binding affinity of fidaxomicin to PDGFR.

CB-Dock2 website: https://cadd.labshare.cn/cb-dock2/php/index.php

Supplementary Figure 2

Fidaxomicin exerted anti-inflammatory effects in LPS-treated CDS-PBMCs but did not affect TGF-β1-mediated fibroblast migration and EMT in intestinal epithelial cells.

(**A**) Cell migration assay. CD-HIFs migrated from the upper chambers of the inserts to the lower chambers containing various reagents in 4 hours. All reagents were added to the lower chambers. TGF-β1 induced cell migration, which was unaffected by fidaxomicin. Mean ± SD. One-way ANOVA was used. Results were pooled from four experiments.

(**B-C**) HPECs in 96-well black clear-bottom plates (165305, Thermo Scientific) were transfected with 5μg/plate COL1A1 promoter-mCherry construct with or without 3pmol/well siRNAs and 0.05μg/well the COL1A1 promoter-mCherry construct via 5μL/well Opti-MEM, 0.3μL/well Lipofectamine 3000, and 100μL/well serum-free DMEM overnight. The transfected HPECs were pretreated with TGF-β1 for 30 minutes. Some groups were treated with fidaxomicin. After 48 hours, Hoechst 33342 nuclear stain (R37605, ThermoFisher) was added. Lionheart LX recorded fluorescence signals. The ratio of red/blue fluorescence indicated COL1A1 promoter activity that reflected EMT activity. Results were pooled from four experiments. Mean ± SD. One-way ANOVA was used.

(**D**) Cytokine ELISA. CDS-PBMCs were treated with CDSE and lipopolysaccharide (LPS) with or without fidaxomicin for 6 hours. The conditioned media were used for ELISA. LPS, but not CDSE, induced IL-8 and TNFα secretion, which was reduced by fidaxomicin. CDSE, but not LPS, mildly induced TGF-β1 secretion, which was unaffected by fidaxomicin. Mean ± SD. One-way ANOVA was used. Results were pooled from 6 CDS patients' PBMCs. Results were pooled from two experiments. Mean ± SD. One-way ANOVA was used.

Supplementary Figure 3

Fidaxomicin did not affect ileal microbiota in SAMP1/YitFc mice.

(**A-C**) The ileal microbiota in 42-week-old SAMP1/YitFc mice and fidaxomicin-treated 42-week-old SAMP1/YitFc mice were compared using shotgun metagenomic sequencing by CosmosID.

(**A**) Alpha diversity by abundance score of ileal microbiota in SAMP1/YitFc mice. The CHAO1 index is an estimator of species richness in the microbiome. Wilcoxon rank sum test: SAMP1/YitFc versus SAMP1/YitFc + Fidaxomicin. The difference was statistically insignificant. Fidaxomicin did not affect the alpha diversity of the ileal microbiota in SAMP1/YitFc mice. Statistical comparison was provided by CosmosID.

(**B**) Beta diversity by relative abundance of ileal microbiota in SAMP1/YitFc mice. The JACCARD measures how similar samples are based on the presence or absence of species without considering their abundance. The Principal Coordinate Analysis (PCoA) plot reflects whether species are present in both samples without considering how many of each species are found. PERMANOVA analysis: SAMP1/YitFc versus SAMP1/YitFc + Fidaxomicin. The difference was statistically insignificant. Fidaxomicin did not affect the beta diversity of the ileal microbiota in the SAMP1/YitFc mice. Statistical comparison was provided by CosmosID.

(**C**) A heatmap of the relative abundance of ileal bacteria of SAMP1/YitFc mice with and without fidaxomicin treatment. The top 12 species are shown. Only three bacterial species are dominant. Fidaxomicin did not consistently affect ileal bacteria species.

n=4 mice per group.

Supplementary Figure 4

Chronic antibiotic treatment did not ameliorate ileal fibrosis in SAMP1/YitFc mice.

(**A**) Experimental plan of mouse ileal fibrosis in SAMP1/YitFc mice. The 20-week oral antibiotic mixture intervention was started at 22 weeks of age when the SAMP1/YitFc mice had early-stage ileitis. Ileal tissues were collected at 42 weeks of age.

(**B**) H&E and MT staining images of ileal tissues. Antibiotic treatment failed to restore normal ileal histology.

(**C**) Ileal histology and fibrosis scores. Mean ± SD. One-way ANOVA was used.

n=6 mice per group. Two rounds of experiments.
