# Supplementary material for: Fidaxomicin Reduces Collagen Expression in Intestinal Fibroblasts Via Platelet-Derived Growth Factor Receptor Beta and Glycogen Synthase Kinase-3 Beta Inhibition
Source: Gastroenterology. Author manuscript; Available in PMC 2026 Jan 21. (PMC12822550; doi:10.1053/j.gastro.2025.04.028)
Supplement: 1 [file NIHMS2137854-supplement-1.pdf]

Supplementary Materials and Methods

10X Genomics Visium Spatial Gene Expression Analysis

Fibrotic ileal tissues from 3 CDS patients (CD02, CD04, and CD05A) were acquired from UCLA Surgical Pathology. They were fixed in formalin and embedded in paraffin at the UCLA Translational Pathology Core Laboratory (TPCL). The paraffin-embedded sections were sent to the UCLA Technology Center for Genomics and Bioinformatics (TCGB) for 10X Genomics Visium spatial RNA sequencing and bioinformatic analysis.

Gene expression analysis was performed using 10X Genomics Visium spatial for formalin-fixed, paraffin-embedded (FFPE) gene expression kit (#1000338) and Novaseq SP with 1 lane of 2 $\times$ 50 SP sequencing. The processed data were presented by 10X Genomics Space Ranger.

The Space Ranger software showed assay performance and gene expression data. For gene expression data, a differential expression test was performed between each cluster and the rest of the sample for each feature. The Log2 fold-change (L2FC) is an estimate of the Log2 ratio of expression in a cluster to that in all other spots. A value of 1.0 indicates 2-fold greater expression in the cluster of interest.

The *P* value measures the statistical significance of the expression difference and is based on a negative binomial test. The *P* value reported here has been adjusted for multiple testing via the Benjamini-Hochberg procedure. The results include lists of up-regulated genes for each cluster with Log2 fold-change and adjusted *P* values.

Fibrotic clusters showed significantly increased collagen (COL1A1, COL1A2, COL3A1, and COL12A1) mRNA expression compared with nonfibrotic clusters.

Super-PRED Target Prediction

The introduction to Super-PRED was excerpted from its website: https://prediction.charite.de/. Super-PRED is a prediction web server for Anatomical Therapeutic Chemical (ATC) code and compound target prediction. The web server's ATC and target predictions are based on a machine learning model using logistic regression and Morgan fingerprints of length 2048. The ATC classification system is used to classify drugs. It was published by the World Health Organization. The classification is based on the therapeutic and chemical characteristics of the drugs.

The ATC code prediction is based on machine learning, using a linear logistic regression model. It is trained on Morgan fingerprints from 1552 different drugs in 233 different level 4 ATC classes. The machine learning model evaluates and scores query compounds, ranking each ATC class and returning the highest-scoring classes.

Cell Migration

Cell migration of intestinal fibroblasts was assessed with a chemotaxis cell migration assay (ECM508, Millipore).[e1,e2] CD-HIFs 1 $\times$ 10^6 cells/mL in serum-free DMEM were added to the inserts. Ten ng/mL TGF-β1 and 10 μM fidaxomicin in serum-free DMEM were added to the 24-well plates. The inserts were loaded to the 24-well plates and incubated at 37°C for 4 hours. The migrated fibroblasts were stained, lysed, and determined by absorbance at 650 nm.

Histologic Evaluations of Mouse Intestinal Tissues

H&E- and Masson's trichrome (MT)–stained microphotographs were recorded at multiple locations and scored blindly by 2 investigators.[e3,e4] The chronic intestinal injury was evaluated in terms of mucosal transformation (0/3/6), round cell infiltration in the lamina propria mucosa (0–3), goblet cell death (0/1), tela submucosa fibrosa (0/1), and granuloma (0/1). The sum of these parameters results in a total score (0–12).[e5] Intestinal fibrosis was scored as normal, mild, moderate, and severe (0–3).[e6]

Calculation of ODA

Histology scores, fibrosis scores, and intestinal gene expression were converted into percentages to evaluate intestinal fibrosis.[e7] ODA is the average value of several commonly reported fibrosis-related and inflammation-related genes.

Shotgun Metagenomic Sequencing of Ileal Bacteria in SAMP1/YitFc Mice

Ileal microbiota samples of 42-week-old SAMP1/YitFc mice with and without fidaxomicin treatment were sent to CosmosID for whole-genome shotgun metagenomic sequencing and data analysis. The sample processing method is proprietary. The CosmosID algorithms identify micro-organisms based on entire genomes. The detailed methodology of shotgun metagenomic sequencing was described in our previous reports.[e8,e9]

Quantitative RT-PCR

Total RNA was extracted using RNeasy (74104, Qiagen) and reverse transcribed into complementary DNA (cDNA) using an iScript cDNA synthesis kit (1708890, Bio-Rad). Quantitative PCRs were run with TaqMan Fast Advanced Master Mix for qPCR (4444556, ThermoFisher) and TaqMan real-time PCR assays (ThermoFisher) in a Bio-Rad CFX384 system.[e10] After normalization with endogenous control genes, relative mRNA quantification was performed by comparing the test and control groups. The fold changes are expressed as $2^{\Delta\Delta Ct}$. Fold-change values >1 indicate a positive or up-regulation. Conversely, fold-change values <1 indicate a negative or down-regulation, and fold-regulation is the negative inverse of the fold-change.

Handling of CDS-PBMCs

CDS-PBMCs (5000 cells/well) were cultured in RPMI1640 with 10% exosome-depleted fetal bovine serum and 100 μg/mL CDSE, with and without 10 μM fidaxomicin. After 6 hours, the cell suspensions were centrifuged at 800*g* for 5 minutes. The cell-free supernatants were collected for human interleukin-8 (DY208), TNFα (DY210), and TGF-β1 (DY240) ELISA from R&D Systems.

Supplementary References

e1. Koon HW, Shih D, Karagiannides I, et al. Substance P modulates colitis-associated fibrosis. Am J Pathol 2010;177:2300–2309.

e2. Wang J, Cheng M, Law IKM, et al. Cathelicidin suppresses colon cancer metastasis via a P2RX7-dependent mechanism. Mol Ther Oncolytics 2019;12:195–203.

e3. Yoo JH, Ho S, Tran DH, et al. Anti-fibrogenic effects of the anti-microbial peptide cathelicidin in murine colitis-associated fibrosis. Cell Mol Gastroenterol Hepatol 2015;1:55–74.e1.

e4. Xu C, Ghali S, Wang J, et al. CSA13 inhibits colitis-associated intestinal fibrosis via a formyl peptide receptor like-1 mediated HMG-CoA reductase pathway. Sci Rep 2017;7:16351.

e5. Kruschewski M, Foitzik T, Perez-Canto A, et al. Changes of colonic mucosal microcirculation and histology in two colitis models: an experimental study using intravital microscopy and a new histological scoring system. Dig Dis Sci 2001;46:2336–2343.

e6. Johnson LA, Luke A, Sauder K, et al. Intestinal fibrosis is reduced by early elimination of inflammation in a mouse model of IBD: impact of a "Top-Down" approach to intestinal fibrosis in mice. Inflamm Bowel Dis 2012;18:460–471.

e7. Xie Y, Fontenot L, Estrada AC, et al. Elafin reverses intestinal fibrosis by inhibiting cathepsin S-mediated protease-activated receptor 2. Cell Mol Gastroenterol Hepatol 2022;14:841–876.

e8. Xie Y, Fontenot L, Estrada AC, et al. Genistein inhibits *Clostridioides difficile* infection via estrogen receptors and lysine-deficient protein kinase 1. J Infect Dis 2023;227:806–819.

e9. Xie Y, Irwin S, Nelson B, et al. Citrulline inhibits *Clostridioides difficile* infection with anti-inflammatory effects. Cell Mol Gastroenterol Hepatol 2025;19:101474.

e10. Koon HW, Shih DQ, Chen J, et al. Cathelicidin signaling via the Toll-like receptor protects against colitis in mice. Gastroenterology 2011;141:1852–1863.e1–3.
